# DNA Double-Strand Breaks Coupled with PARP1 and HNRNPA2B1 Binding Sites Flank Coordinately Expressed Domains in Human Chromosomes

**DOI:** 10.1371/journal.pgen.1003429

**Published:** 2013-04-04

**Authors:** Nickolai A. Tchurikov, Olga V. Kretova, Daria M. Fedoseeva, Dmitri V. Sosin, Sergei A. Grachev, Marina V. Serebraykova, Svetlana A. Romanenko, Nadezhda V. Vorobieva, Yuri V. Kravatsky

**Affiliations:** 1 Department of Genome Organization, Engelhardt Institute of Molecular Biology, Moscow, Russia; 2 Laboratory of Proteomics, Shemyakin-Ovchinnikov Institute of Bioorganic Chemistry, Moscow, Russia; 3 Laboratory of Diagnostic Proteomics, Orekhovich Institute of Biomedical Chemistry, Moscow, Russia; 4 Department of Molecular and Cellular Biology, Institute of Molecular and Cellular Biology, Novosibirsk, Russia; Edinburgh Cancer Centre, United Kingdom

## Abstract

Genome instability plays a key role in multiple biological processes and diseases, including cancer. Genome-wide mapping of DNA double-strand breaks (DSBs) is important for understanding both chromosomal architecture and specific chromosomal regions at DSBs. We developed a method for precise genome-wide mapping of blunt-ended DSBs in human chromosomes, and observed non-random fragmentation and DSB hot spots. These hot spots are scattered along chromosomes and delimit protected 50–250 kb DNA domains. We found that about 30% of the domains (denoted forum domains) possess coordinately expressed genes and that PARP1 and HNRNPA2B1 specifically bind DNA sequences at the forum domain termini. Thus, our data suggest a novel type of gene regulation: a coordinated transcription or silencing of gene clusters delimited by DSB hot spots as well as PARP1 and HNRNPa2B1 binding sites.

## Author Summary

The study of chromosomal domains is hampered both by the complexity of chromosomal architecture and by the lack of experimental approaches for identification of structural and functional elements in chromosomes. The primary nucleosomal level in organization of chromosomes was detected by digestion of chromatin with exogenous nucleases. Many years ago, we detected 50–250 kb chromosomal domains that are released when genomic DNA is prepared under mild conditions, now known as forum domains. We assumed that this large-scale fragmentation of chromosomes by endogenous nucleases reflected some higher-order level of organization in chromosomes. However, both the existence of large 50–250 kb protected regions and short regions possessing hot spots of DNA breakage might reflect specific features in organization of chromosomes. Here, we used a procedure for genome-wide mapping of terminal regions of forum domains in human chromosomes. Our results show that hot spots of DNA breaks are not randomly distributed along chromosomes, that adjacent to these regions are binding sites of PARP1 and HNRNPA2B1, and that forum domains contain coordinately expressed gene clusters.

## Introduction

DNA double-strand breaks (DSBs) in the human genome have been studied for many years, for two main reasons. First, DSBs, aberrant repair, and further chromosomal damage lead to massive genomic rearrangements [1]–[4]. These can drive the development of cancer by, for example, deleting tumor suppressor genes, amplifying genes promoting tumorigenesis, and causing oncogenic fusion of regulatory regions with other genes [5]. Second, cytogenetic and molecular studies of chromosomal fragile sites have found that these sites are prone to damage upon replication, because replication initiation sites are scarce or replication fork movement is impaired [3], [6]–[9]. In the FRA16C region, origin density is higher than in the entire genome, but replication forks stall at AT-rich sequences. Under replication stress the region fails to activate additional origins of replication [10]. Transcription of very large (∼1 Mb) human genes that takes more than one cell cycle to complete may also cause DSBs, when collisions occur between transcription and replication machinery [11]. DSBs increasingly are also recognized as preferential targets of oncogene-induced DNA damage [12]. The data suggest that unknown chromosomal regulatory mechanisms may be involved in replication, transcription, and DSB formation in various normal cell types [9], [11], [13]. These could be affected and responsible for the massive genomic rearrangements seen in cancer cells [4], [14].

Chromosomal integrity is challenged by both exogenous (e.g., chemicals, ultra-violet light, radiation, viral infection, or high-salt environments) and endogenous (e.g., oncogenes, replication–transcription collision, oxidative stress factors, or programmed DSBs initiated by meiotic recombination) influences [11], [12], [15]–[19]. The entire repertoire of causes of DSBs in human cells is not known. In attempts to isolate intact chromosomal-length DNA within DNA–agarose plugs, we previously found that up to 10% of chromosomal DNA is excised by some unknown mechanism, even with very quick isolation procedures. This DNA migrates mainly in the 50–250 kb range in pulsed-field agarose gels (PFGs), and represents all chromosomal regions. This phenomenon was observed in various eukaryotes, including different types of human cells and *Drosophila*, plant, and yeast cells. The DNA domains within this 10% of chromosomal DNA were named “forum domains” [20], [21] Recently, we performed genome-wide profiling of forum domains in *Drosophila* S2 cells and found that they are separated by hot spots of DSBs. Frequently, forum domain termini (FT), where individual DSBs reside, were found to correspond to regions of intercalary heterochromatin (I-HC) known as fragile and late replicating regions in *Drosophila* polytene chromosomes [22], [23]. Interestingly, in *Drosophila* chromosomes, the clusters of co-expressed genes [24], [25] and the main HOX gene complexes were found inside separate forum domains [22].

The non-random excision of forum domains from chromosomes and the correspondence of DSB hot spots to I-HC sites reminded us of Laird's old supposition that human fragile sites correspond to the I-HC detected in *Drosophila* [26], [27]. These data and the data on extensive DSBs described in cancer cells and on coordinated expression of genes within forum domains suggested the necessity of whole-genome mapping of DSBs in human cells in order to elucidate the nature of the DNA sequences bordering the large chromosomal domains in human cells.

Here we report the results of genome-wide profiling of DSBs in human chromosomes from cultured HEK 293T cells. Our deep-sequencing data indicate a non-random distribution of DSBs that have blunt ends and that define mainly 50–250 kb forum domains that are protected from the breaks. Transcription profiling demonstrates that some domains contain coordinately expressed gene clusters, suggesting novel regulation of transcription inside large genomic regions delimited by hot spots of DSBs. A clue to a mechanism is provided by our finding that forum termini bind specifically the known transcriptional regulator PARP1, which is involved in chromatin organization and transcriptional regulation, and HNRNPa2B1, a nuclear RNA processing factor. Our data also suggest that the mapped breaks could correspond to fragile sites in human chromosomes, and indicate that our technique can be used to study fragile chromosomal sites in many types of human cells.

## Results

### The rapid amplification of forum domains termini (RAFT) procedure for 454 sequencing, and the genome-wide mapping of FT

**Figure 1:**
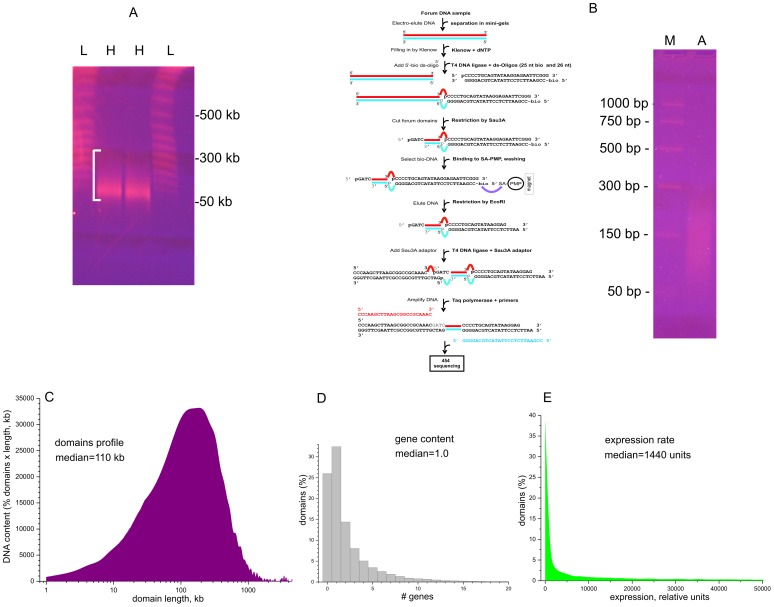
Isolation and amplification of terminal regions of forum domains and general properties of domains. (A) Electrophoretic separation of DNA from DNA–agarose plugs (25 sec pulses). L, lambda ladder; H, forum domains DNA preparation from HEK 293T cells. White bracket shows the 50–300-kb region. (B) RAFT procedure for amplification of whole-genome FT and deep sequencing. The gel shows the separation of the final RAFT probe. A, amplified RAFT DNA; M, PCR marker. (C) Profile of domains observed after mapping of FT. (D) Gene content of forum domains determined using the data from ftp://ftp.ncbi.nlm.nih.gov/genomes/H_sapiens/Assembled_chromosomes/gbs/hs_ref_GRCh37.p5_chr.gbs.gz. (E) Plot of expression levels within forum domains determined using mRNA-seq data from IMR90 cells (GEO accession number GSM438363).

To study hot spots of spontaneous DSBs causing forum domain excision, we isolated DNA samples in a solid phase, as described [20], [21]. The procedure prevents the random hydrodynamic shearing of very long DNA molecules in solution. Human HEK 293T cells were included inside 0.5% low-melt agarose at 42°C in DMEM. After incubation for 2–5 min on ice, the solid agarose plugs were placed in Petri dishes with a solution containing 0.5 M EDTA (pH 9.5), 1% sodium lauryl sarcosine, and 1–2 mg of proteinase K solution per mL. The samples then were incubated for 40–48 h at 50°C. Figure 1A shows the pattern of migrating forum domains under fractionation in the PFG. The migrating DNA was detected mainly in the 50–250 kb region. For genome-wide mapping of DSBs, we isolated and amplified forum domain termini (FT) using the RAFT approach. This is schematically illustrated in Figure 1B (see Text S1 for details). The amplified DNA was used for 454 sequencing and in fluorescence in situ hybridization (FISH) on human chromosomes. The RAFT reads were processed as described in Text S1 and in the databases (GEO accession number GSE35065). The mapping was performed using the human genome assembly of February 2009 (GRCh37/hg19). An FT is defined as a DNA fragment confined by individual DSBs and Sau3A site and usually is from 50 to 300 bp in length (Figure 1B).

Figure 1C shows that the mapped domain sizes range from 1 kb to 3 Mb, with a median of about 110 kb, consistent with the PFG separation results (Figure 1A). The data are in agreement with the results for non-random fragmentation in the *Drosophila* genome [22]. Hot spots of DSBs are scattered along human chromosomes (Figures S1, S2, S3). Genome-wide analysis revealed that about one-third of domains separated by hot spots of DSBs include from three to seven genes (Figure 1D). This is to be expected, because the domains are rather long. The analysis of the domains' transcription strength using the available mRNA-seq data (GEO Accession Number GSM438363, IMR90 cells) indicates that most forum domains (up to 90%) are silent or transcribed at low levels, with only a small number actively transcribed (Figure 1E). A more detailed analysis of forum domain gene expression patterns is presented below.

### FISH of the RAFT preparation

**Figure 2:**
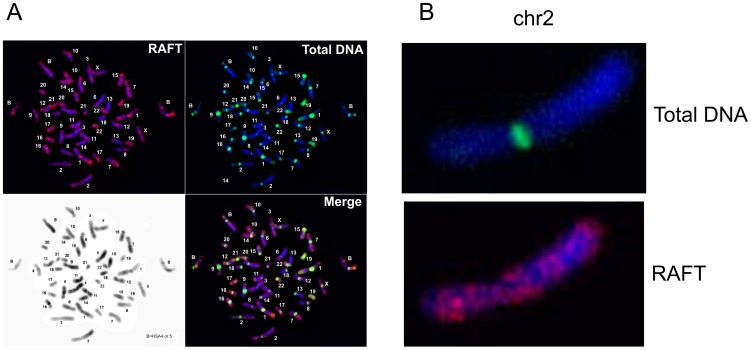
Fluorescent *in situ* hybridization of the RAFT probe. (A) Hybridization patterns of the RAFT probe and total DNA. (B) Hybridization patterns inside chr2 shown in more detail.

FISH analysis (Figure 2) indicates that the RAFT probe, which corresponds to less than 0.2% of the genome, nevertheless gives much more diverse signals, mainly for G-positive bands, than total DNA isolated from HEK 293T cells, which marks mostly pericentromeric heterochromatic blocks, even though over two-thirds of the human genome has been identified as dispersed transposable elements and low-complexity repeats [28]. The result for the RAFT probe was expected, insofar as we labeled FT produced by DSBs that are scattered along chromosomes (Figure 2B). However, we also detected RAFT signals in the ribosomal genes on all acrocentric chromosomes bearing clusters of rDNA (chr13, chr14, chr15, chr21, chr22). Because there are significantly fewer copies of rDNA than there are SINEs and LINEs, the emergence of these signals was clearly not accidental. In addition, intense signals not matched with G-bands were found on chromosome chr17 and in the distal region of chr1p.

### Hot spots of spontaneous DSBs in chromosomes and their relation to fragile sites

**Figure 3:**
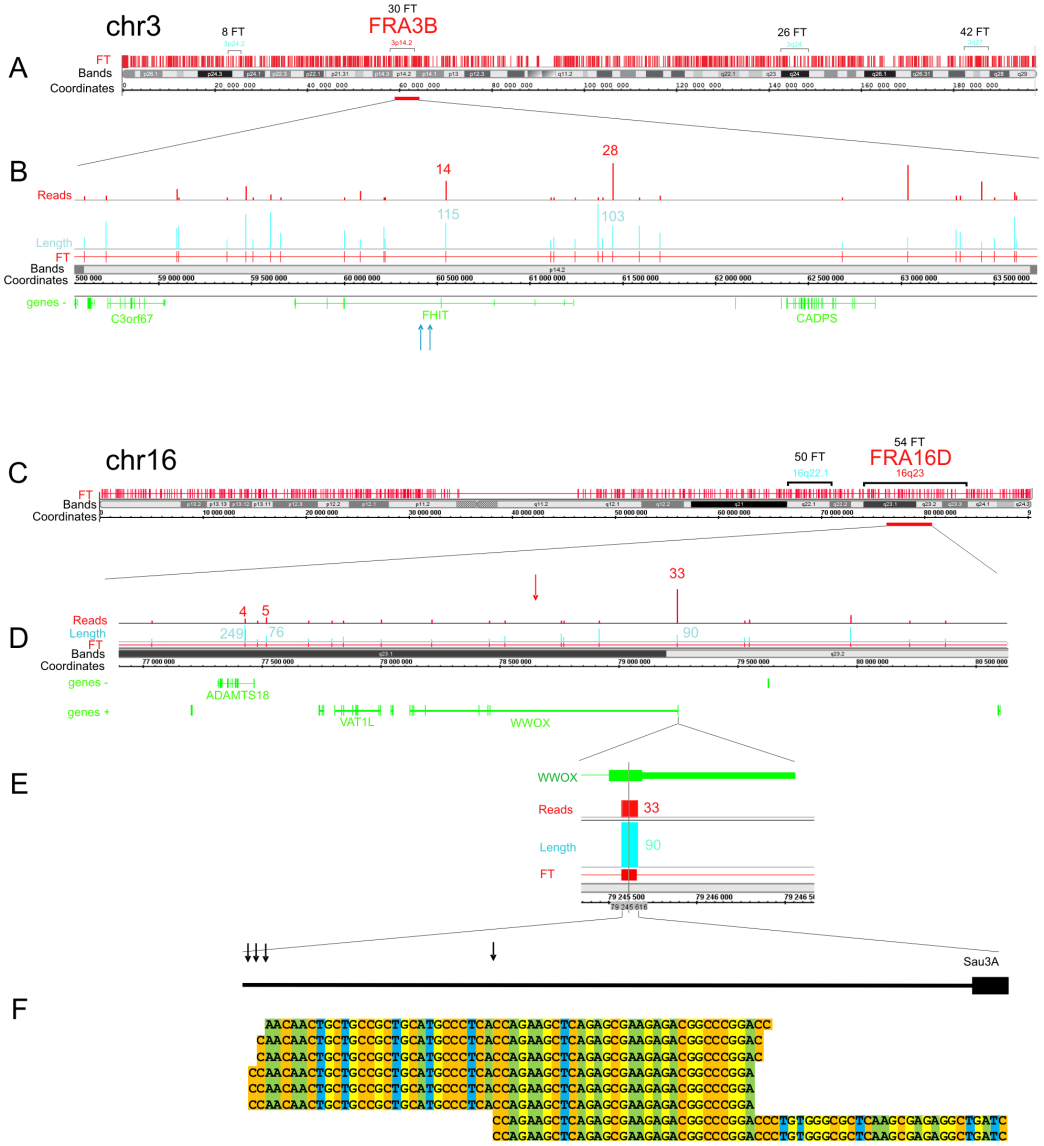
Overviews of chromosomes 3 and 16, which contain the FRA3B and FRA16D regions. The frequently and less frequently observed CFS detected in leukocytes are shown in red and blue, respectively. The Integrated Genome Browser (Affymetrix) was used (http://bioviz.org/igb/). (A) FT barcode in the overview of chr3. (B) FRA3B region that contains the *FHIT* gene. Mapped FT are shown. The length and number of reads are indicated. Blue arrows indicate the positions of the previously described DNA breaks inside *FHIT* in tumor cells (GenBank: AF020610.1 and U85047.1). (C) FT barcode in the overview of chr16. (D) FRA16D region containing the *WWOX* gene. Mapped FT are shown. The length and number of reads are indicated. The previously mapped breaks inside the minisatellite repeat in the *WWOX* gene are indicated by the red arrow (GenBank: U85253.1). (E) The length and number of FT reads inside the 3′ exon are indicated. (F) Mapped cut sites inside the 3′ exon are indicated by black arrows. Some of the corresponding RAFT reads are shown at the bottom.

Figure 3 shows the pattern of FT on overviews of chromosomes 3 and 16, where the most sensitive fragile sites in human leukocytes were described [29] (the patterns of mapped FT in other chromosomes are shown in Figures S1, S2, S3). The common fragile sites (CFSs) in the regions of the very large tumor suppressor genes *FHIT* and *WWOX* (FRA3B and FRA16D, respectively) have mapped DSB hot spots in HEK 293T cells. In the approximately 1500 kb fragile histidine triad gene, *FHIT*, eight FT were mapped in the gene itself and ten FT close to it. Monte-Carlo simulations indicated that the probability of the random occurrence of this number of FT consisting of overlapping reads is very low (p<10^−7^), both for FHIT itself and the whole region. Details are provided in Text S1. The gene is a known target in FRA3B (Figure 3A and 3B). In the approximately 1115 kb *WWOX* gene, a target in FRA16D, seven FT corresponding to DSB hot spots were mapped (Figure 3C and 3D). The probability of the random occurrence of the detected amount of FT in the *WWOX* gene is less than 10^−7^, or 0.00001%. One FT was mapped in the coding region of the 3′ exon. The corresponding individual DSBs inside this FT occur at four positions within a 30 bp region of the exon (Figure 3E and 3F).

To check the frequencies of DNA breaks in this region, we used quantitative real-time PCR across the FT (Figure S4 and Figure S5). We found that a roughly 1 kb DNA fragment including the FT region contains palindromes and a Z-DNA region. The real-time PCR experiments across the FT strongly support the existence of DNA breaks in this particular region. In DNA preparations isolated in solution from HEK 293T cells by a procedure that simulates the isolation of forum domains in agarose plugs (see Text S1), up to 20% of DNA molecules spanning the region are damaged compared with DNA preparations isolated immediately after precipitation of cells (Figure S5). The break sites, which lie within a rather short region of this FT, were visualized by the RAFT procedure and 454 sequencing (Figure 3F). The data suggest that the breaks occur at specific sites during a very short incubation of cells in DMEM. However, the nature of the enzyme that produces the DSBs is unknown.

To determine if the *WWOX* 3′ exon sequence containing FT could be fragmented as part of a plasmid after transfection into HEK293T cells, we attempted to clone the amplified 1090 bp fragment (Figure S4), but our attempts were unsuccessful. This fragment, and a shorter one of 490 bp (shown in Figure S4) that includes the central part of the FT, escaped cloning in pGL-3-Enhancer, pGEM-T-easy, and pUC12 vectors. We conclude that this region of the *WWOX* gene cannot be cloned in *E. coli*. The presence of palindromes and the Z-DNA region might hamper the replication of this DNA in *E. coli* at 37°C, while both *Taq* and *Pfu* polymerase successfully synthesize it at 72°C. It is possible that this region is also a “strong stop” for human DNA polymerases, which may explain why we found it to be a hot spot for DSBs.

**Figure 4:**
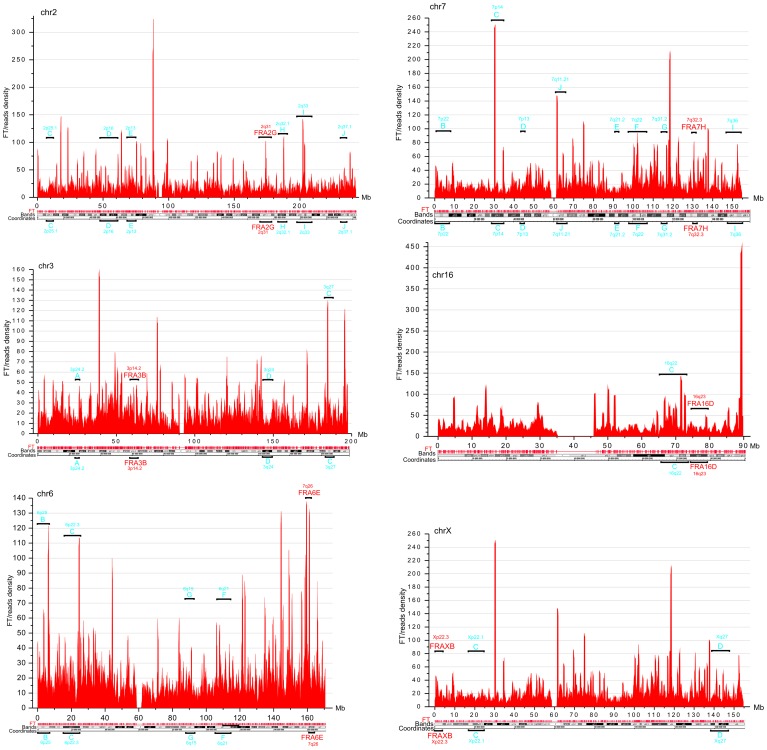
FT density across chromosomes 2, 3, 6, 7, 16, and X, which possess the frequently and less frequently observed CFS in leukocytes. Window = 500 kb; step = 100 kb. The frequently and less frequently observed CFS detected in leukocytes are shown in red and in blue, respectively.

DNA fragility occurs in late replicating regions. It requires nucleotide sequences that are prone to forming secondary structures that may impair replication fork movement [10], [29], and/or a scarcity of replication initiation sites in these regions. The latter reflects cell-type-specific replication programs, which are established by unknown mechanisms [9], [13]. Thus, we cannot expect that the pattern of fragile sites described in leukocytes will be exactly the same in HEK 293T cells. In fact, our data support the conclusion that CFSs are cell-type specific (9). The observed profiles of FT density in HEK 293T cells (Figure 4) in chromosomes 2, 3, 6, 7, 16, and X, in which frequently observed CFSs in leukocytes were described [29], are mainly different from that in leukocytes [9]. Nevertheless, in chromosome 6 and others, there was a clear correlation between the peaks of FT density in HEK 293T cells and the previously described frequently and less frequently observed CFSs in leukocytes, (Figure 4). We conclude that the mapped FT possessing spontaneous DSBs could correspond to fragile sites in human chromosomes. Future mapping of FT in different cell types, with direct comparison with replication profiles (e.g., in the lymphoblastoid and fibroblastic cells), will be of interest.

### Analysis of expression profiles in forum domains

**Figure 5:**
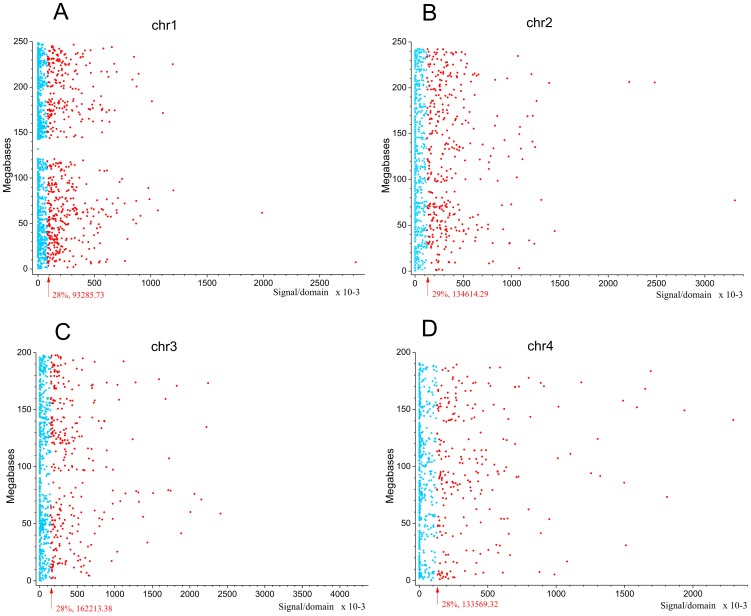
Expression levels inside forum domains in chr1, chr2, chr3, and chr4. The data for expression in HEK293T cells (microarray data using Affymetrix Human Exon 1.0 ST expression arrays, http://www.affymetrix.com/estore/browse/products.jsp?navMode=34000&productId=131452&navAction=jump&aId=productsNav#1_1, wgEncodeEH002692_2) were used. The median values of transcription levels in coding regions (representing exon array signals) within a particular forum domain were used, and the result was plotted according to the position of the domain in its chromosome. (A–D) Expression levels inside forum domains in the largest chr1, chr2, chr3, and chr4. The arrows indicate the position of the average expression level of forum domains in a particular chromosome. The value to the right of the arrow indicates the proportion of forum domains in a chromosome that is more highly expressed. Domains with expression levels lower or higher than the average expression level are shown by the blue and red spots, respectively.

Next we asked whether the observed pattern of non-randomly dispersed spontaneous DSBs has some relation to the expression patterns of genes, because clusters of co-expressed genes were observed in *Drosophila melanogaster* forum domains [22]. We used the search of mRNA expression profiles in HEK293T cells (microarray data, wgEncodeEH002692_2) inside forum domains along human chromosomes. The median values of transcription levels in coding regions (representing exon array signals) within a particular forum domain were used, and the result was plotted according to the position of the domain in its chromosome. We observed that the proportion of actively expressed forum domains mainly varies at value of about 30% in different chromosomes (Figure 5A–5D and Figures S6, S7, S8, S9, S10, S11, S12). Most domains are silent or expressed at very low levels. The mRNA transcription profiles are in agreement with the genome-wide data shown in Figure 1E, demonstrating that most forum domains (about 70%) are silent or transcribed at low levels, whereas only a small number are actively transcribed. The average expression levels per forum domain in different chromosomes are shown in Figure S12.

**Figure 6:**
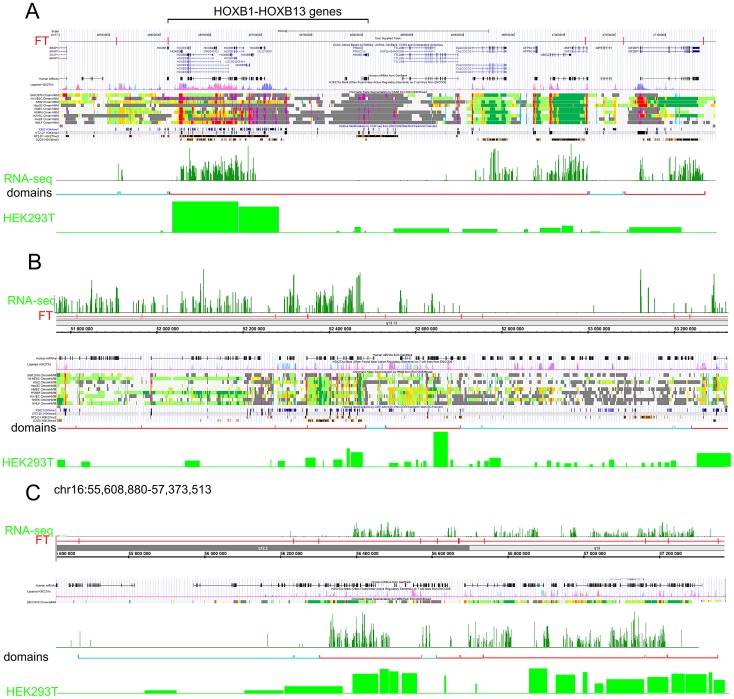
Coordinated expression inside forum domains. The UCSC Genome Browser, Human Feb. 2009 (GRCh37/hg19) Assembly was used. UCSC genes, human mRNAs from GenBank, the H3K37Ac mark from Encode, chromatin state segmentation by HMM from Encode/Broad, and some histone modifications by ChIP-seq from Encode are indicated (Ernst et al., 2011). The “RNA-seq” lane corresponds to the expression of mRNAs in IMR90 cells (GEO accession number GSM438363). The “Domains” lanes indicate the forum domains containing silent or weakly expressed genes (blue brackets), or domains possessing actively transcribed genes (red brackets). “HEK293T” lanes correspond to the expression of mRNA in HEK293T cells (microarray data using Affymetrix Human Exon 1.0 ST expression arrays, http://www.affymetrix.com/estore/browse/products.jsp?navMode=34000&productId=131452&navAction=jump&aId=productsNav#1_1, wgEncodeEH002692_2). (A) Region of chr17 that includes the *HOXB* gene cluster and contains active and silent domains. (B) Region of chr12 that includes active, low expressing, and silent domains. The leftmost domains are actively transcribed in IMR90 cells, but are low expressed in HEK 293T cells. (C) Region of chr16 that includes active and silent domains in IMR90 cells, and active and low expressing domains in HEK293T cells.

Figure 6A–6C and Figures S13 and S14 show some examples of neighboring transcriptionally active and silent forum domains in HEK293T cells and in IMR90 cells in different chromosomes, including the regions comprising the main *HOX* gene clusters. The *HOXB* gene cluster is co-expressed with several other genes inside the 413 kb domain (Figure 6A). The active chromatin marks determined in nine human cell types [30] and transcribed regions in this area of chr17 are delimited by FT corresponding to hot spots of DSBs. The data shown in Figure 6A indicate that the two FT that delimit a gene cluster containing HOX-B genes are located on the borders of actively transcribed regions. There are some differences in expression profiles inside forum domains in HEK293T and in IMR90 cells, e.g., the leftmost domains shown in Figure 6B are active in IMR90 cells, while they are low expressed in HEK293T cells. Similar results were observed in forum domains containing the *HOXA*, *HOXC*, and *HOXD* genes (Figures S13 and S14). These data suggest that transcription is regulated through unknown mechanisms over big chromosomal regions delimited by hot spots of chromosomal breaks. It seems likely that DSB hot spots are relevant to transcriptional control as well as replication. The data on collisions between replication and transcription complexes leading to CFS instability [11] are clearly consistent with this conclusion.

The data on circular shifting by random value of gene median expression levels described in Text S1 strongly indicate that the genes in the same forum domain differ much less in their expression levels compared to genome-wide differences (p-value<0.0001). A mechanistic link between DSBs and transcription patterns is independently supported by the fact that, in *Drosophila* chromosomes, silenced Pc-domains are located within forum domains [22].

### PARP1 and HNRNPA2B1 bind specifically to FT sequences *in vitro*

**Figure 7:**
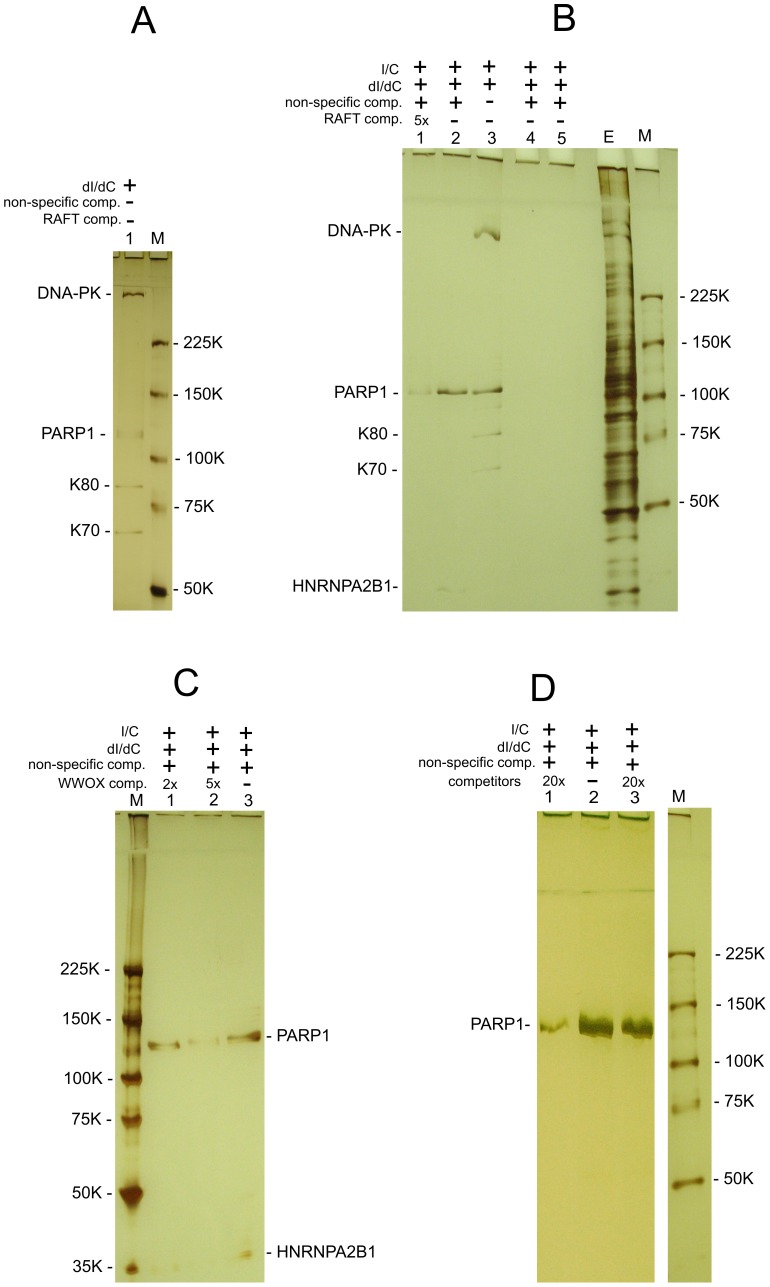
Selection of proteins binding with RAFT preparations and with the individual FT from *WWOX* gene. All indicated proteins were identified by mass spectrometry as described in Text S1. (A) Binding of nuclear proteins with biotinylated RAFT preparations (0.4 µg) was revealed by the use of SA-PMP (see Extended Experimental Procedures in Text S1). Poly[d(I)/d(C)] competitor DNA (dI/dC) and poly[(I)/(C)] competitor RNA (I/C) were used. E, extract of nuclear proteins; M, marker. Proteins were separated by use of 5% PAGE. (B) Binding of nuclear proteins to biotinylated RAFT preparations. dI/dC and I/C non-specific competitors, and PCR-amplified non-specific competitor and RAFT-specific competitor DNAs, both synthesized using Taq polymerase, were used. The non-specific DNA competitor efficiently eliminates end-binding proteins. E, extract of nuclear proteins; M, marker. Lanes 4 and 5 correspond to experiments with single-stranded or double-stranded biotinylated oligos, respectively, that were used for amplification of the RAFT probes. Proteins were separated by use of 5–18% PAGE. (C) Binding of nuclear proteins to biotinylated 1050-bp *WWOX* FT preparations (see Figure S4). dI/dC and I/C non-specific competitors, and PCR-amplified non-specific competitor and *WWOX-*specific competitor DNAs, both synthesized using Taq polymerase, were used. E, extract of nuclear proteins; M, marker. Proteins were separated by use of 5–18% PAGE. (D) Binding of nuclear proteins to biotinylated RAFT preparations. dI/dC and I/C non-specific competitors, and PCR-amplified non-specific competitor and RAFT-specific competitor DNAs, both synthesized using Taq polymerase, were used. Lanes 1 and 3 correspond to experiments with 20x excesses of RAFT preparation lacking the biotin label (8 µg) or total human DNA (8 µg) digested with Sau3A enzyme, respectively (competitors). Lane 2 corresponds to the experiment with no specific competitors; M, marker. Proteins were separated by use of 5% PAGE.

The co-expression data suggest that there are unknown mechanisms silencing or activating the transcription of multiple genes inside the large chromosomal domains delimited by DSB hot spots. We hypothesized that some master regulatory proteins should control gene expression in the 50–250 kb and even larger forum domains. We performed a search for nuclear proteins that could specifically bind to FT. We developed a technique that is based on the gel-retardation method, but uses biotinylated DNA preparations and purification of binding proteins on Streptavidin MagneSphere Paramagnetic Particles (SA-PMP, Promega) (for details, see Text S1). Initially, we used RAFT preparations that are a complex mixture of whole-genome-amplified short chromosomal regions attached to DSB hot spots (Figure 7). Using poly(dI-dC), the most widely used non-specific competitor, and 5% PAGE, we observed that four proteins bound to the RAFT preparation: DNA-PK, PARP1, K80, and K70 (Figure 7A). Ku70–Ku80 proteins recruit DNA-PK to DNA ends. These three proteins form complexes that are involved in non-homologous end joining (NHEJ), the most prevalent DSB repair mechanism in mammalian cells, by which DNA DSBs are ligated independently of their sequences [31]–[34]. As any linear, synthetic double-stranded DNA can bind to these proteins non-specifically, in subsequent experiments we used a non-biotinylated, linear, PCR-amplified DNA fragment of pGL3-Enhancer vector as a non-specific competitor to provide a large molar excess of double-stranded DNA ends. In the experiments using this non-specific DNA competitor to eliminate end-binding proteins, and fractionation through 5–18% gradient polyacrylamide gels, we observed only two proteins binding to the DNA sequences at DSBs in different genomic regions of RAFT samples: poly(ADP-ribose) polymerase 1 (PARP1) and heterogeneous nuclear ribonucleoprotein A2/B1 (HNRNPA2B1) (Figure 7B). The observed binding was not due to the oligos used for amplification of the RAFT probes (lanes 4 and 5 in Figure 7B).

The specificity of PARP1 and HNRNPA2B1 binding with FT sequences was confirmed by using RAFT preparations lacking the biotin label as competitor. As expected, this abolished the binding of both proteins (lane 1 in Figure 7B). The 20x excess of total human DNA did not interfere with the binding of the proteins (lane 3 in Figure 7D), indicating that RAFT preparation is essentially enriched in sequences binding PARP1 and HNRNPA2B1. Independently, both proteins were also found to bind specifically in the selected conditions to the FT at the 3′ exon of the *WWOX* gene (Figure 7C). Interestingly, the molar ratio of each individual FT sequence in the complex mixture of thousands of whole-genome FT in 0.4 µg of the RAFT preparations is very low, but all together they bind approximately the same amounts of PARP1 and HNRNPA2B1 proteins as 0.4 µg of individual FT from the *WWOX* gene does (Figure 7B and 7C). Consequently, the majority of FT in the RAFT preparation bind these two proteins. The data support our hypothesis that important regulators of transcription and chromatin structure bind at FT regions very close to DSB hot spots, but not to DNA ends.

### Replication stress or heat shock treatment enhances DNA breakage at FT sequences

**Figure 8:**
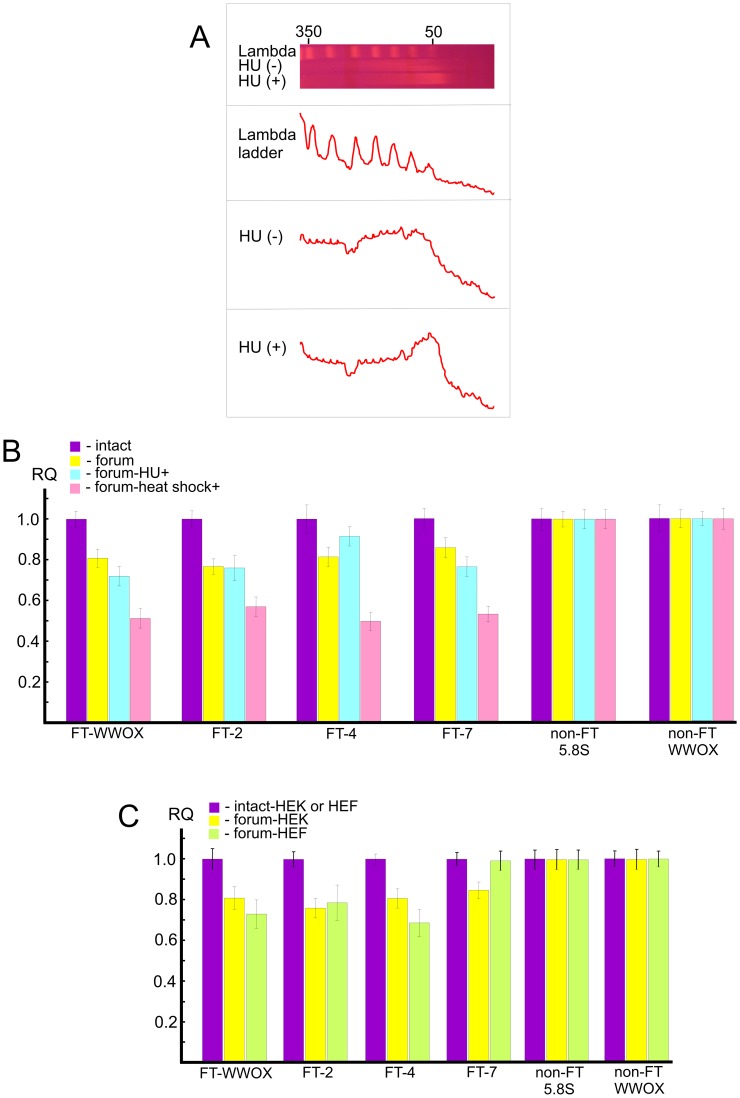
Analysis of hot spots of DSBs by quantitative PCR across several FT. (A) PFG- separation of forum domains isolated from HEK 293T cells untreated or treated by HU, as described in Text S1. The run was performed under 25 sec pulses for 29 h. The profiles of λ–ladder and forum domains are presented. The values above the gel indicate the length of DNA fragments in kb. (B) PCR experiments using untreated HEK 293T cells or cells incubated in the presence of 0.2 mM HU for 18 h or heat shock-treated cells. As a control, two regions devoid of FT (non-FT regions) from the 5.8S ribosomal gene or the *WWOX* gene were used (all primers are indicated in Table S1). For details, see Extended Materials and Methods in Text S1. The results of four independent experiments are shown. RQ – relative quantities to undamaged DNA. (C) HEK 293T or HEF cells were used for isolation of intact DNA or forum domain preparations, and real-time PCR experiments were performed as described in the Supporting Information. As a control, two non-FT regions from the 5.8S ribosomal gene or the *WWOX* gene were used (all primers are indicated in Table S1). The results of four independent experiments are shown. RQ – relative quantities to undamaged DNA.

In order to study independently a link between FT and the chromosomal fragile sites, we used HEK 293T cells grown for 18 h under replication stress induced by hydroxyurea (HU) (for details, see Text S1). Separation of forum domain preparations isolated from HU-treated and non-treated cells in the PFG revealed that replication inhibition leads to a shift of domains profile to the 50 kb region, whereas domains isolated under the same condition from non-treated cells are mainly larger (Figure 8A). Thereafter, under replication stress more DNA molecules are damaged, and this affects the profile of forum domains in the PFG. This fact argues in favor of the conclusion that at least some part of FT defining forum domains corresponds to fragile sites in chromosomes.

Quantitative PCR revealed that the portion of DNA molecules containing DSBs at two FT (FT-WWOX and FT-7) from the four FT analyzed was increased in the HU-treated cells (Figure 8B). FT-4 was less sensitive to the replication stress, whereas FT-WWOX was very sensitive to it. Up to 27% of DNA molecules corresponding to this small 182-bp region were damaged in the HU-treated cells. These data independently support our supposition about a possible relationship between FT and fragile sites that was drawn from the profiles of FT density along chromosomes (Figure 4).

Profiling FT along chromosomes also revealed a correspondence between transcription patterns and hot spots of DSBs. To address the question of whether global gene expression changes could interfere with DNA breakage at FT regions, we analyzed the same FT set in heat shock-treated cells (see Text S1). Interestingly, we observed more profound DNA breakage for all FT tested in heat shock-treated cells than in HU-treated ones. Figure 8B shows that up to 50% of DNA molecules from these regions were damaged, as tested by quantitative PCR across several FT. This was not due to shearing of DNA upon isolation, because two non-FT regions (5.8S ribosomal gene and non-FT region located 19.7 kb upstream from FT in the 3′ exon of the *WWOX* gene) were found to be undamaged. FT-4, which was slightly affected by replication stalling induced by HU, exhibited a high sensitivity to heat shock treatment. The results independently demonstrate a strong relationship between organization of transcription patterns and hot spots of DSBs.

The observed relationships of FT regions with replication and transcription events suggest that at least some part of these hot spots of DSBs could be located in the same regions in different cell types due to stability of some higher-order chromosomal structures. The irrefragable answer could be found in the genome-wide profiling of FT in several cell types, which we currently are performing. In the present study, we used only quantitative PCR across several FT in one more cell line: human embryonic lung fibroblast (HEF) cells. Figure 8C shows that among the four FT tested, three FT are present in both HEK 293T and HEF cells. FT at the 3′ exon of the *WWOX* gene and FT-4 are even more sensitive in fibroblasts than in HEK 293T cells, where they originally were detected. At the same time, FT-7 is absent in the HEF cells. Thus, not all FT retain their position in different cell types. The data suggest the functional significance of FT regions in differentiation.

### PARP1 and HNRNPA2B1 bind specifically to FT sequences *in vivo*

**Figure 9:**
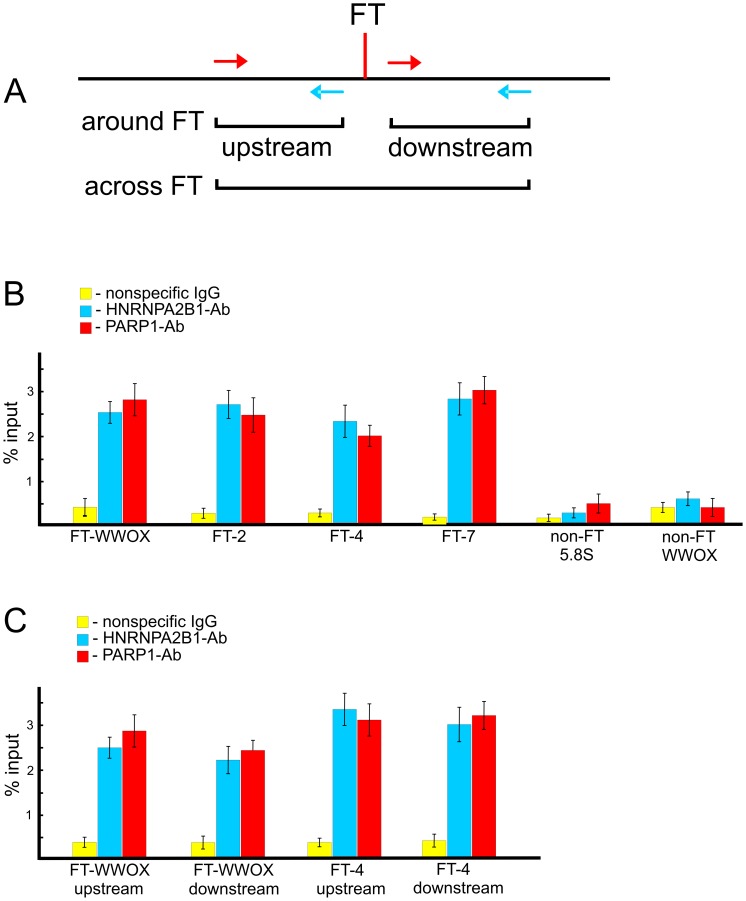
ChIP experiments using antibodies to PARP1 or to HNRNPA2B1. (A) Scheme illustrating the PCR strategies used for amplification of DNA fragments across FT or around it. (B) Results of PCR across four FT and two non-FT regions (from the *WWOX* gene and the 5.8S ribosomal gene) using immunoprecipitated DNA. Primers used are shown in Table S1. Percentage of input DNA is indicated, n = 4. (C) Results of PCR around two FT. Primers used are shown in Table S2. Percentage of input DNA is indicated, n = 4.

To test whether PARP1 and HNRNPA2B1 bind at FT regions in live cells, we performed chromatin immunoprecipitation (ChIP) using PARP1 and HNRNPA2B1 antibodies and quantitative PCR. In our *in vitro* binding experiments (Figure 7) we used RAFT preparations containing a genome-wide mixture of FT comprising 50–300 bp DNA fragments each confined by a nucleotide at DSB and Sau3A sites (Figure 1B). The *in vitro* binding data show that PARP1 and HNRNPA2B1 bind to nucleotide sequences in the regions that are adjacent to FT. If both proteins reside at forum termini when DNA is undamaged, we could detect such binding using PCR across FT (Figure 9A). Alternatively, if the proteins bind only when DNA is broken at the FT region, we have to use another set of primers that define shorter regions on both sides around the FT (Figure 9A). The results of the ChIP experiments are shown in Figure 9B and 9C. The primers used are indicated in Tables S1 and S2. We observed that all four FT tested bind PARP1 and HNRNPA2B1. Two non-FT regions used in the analysis did not bind the proteins. These data strongly indicate that immunoprecipitated chromatin fragments are enriched by both the FT sequences and by PARP1 and HNRNPA2B1. We observed the *in vivo* binding of the proteins using both PCR approaches. The data shown in Figure 9C are in agreement with the observed binding of PARP1 and HNRNPA2B1 with RAFT preparations containing regions around FT (from sites of DSBs to Sau3A sites). In the ChIP experiments using the first variant of PCR, only undamaged DNA molecules were amplified (Figure 9B). This result strongly suggests that PARP1 and HNRNPA2B1 reside on borders of forum domain termini that are intact. Such a distribution of the proteins shapes specific kinds of domains in chromosomes. Nevertheless, our results do not mean that PARP1 and HNRNPA2B1 bind only at FT regions. The ChIP-seq experiments that we currently are performing could clarify the question in the near future.

## Discussion

### Spontaneous DNA fragmentation could reflect the existence of physiological mechanisms for large-scale DNA breakage

Integrity and fragmentation are both natural states of chromosomal DNA. For example, extensive DNA fragmentation is observed in elongating spermatids during their normal chromatin remodeling [35], [36]. Local DNA breaks are produced during somatic V(D)J recombination in primary lymphoid tissue, during meiotic recombination, and in transpositions in somatic and germ line cells. Transient widespread DNA fragmentation has also been described in various pathological processes in nerve cells [37]. Extensive DNA breakage (“chromothripsis”) was described in cancer cells [4]. Complex genomic rearrangements, probably involving a replication-based mechanism, were described in genomic disorders [14]. The formation of the macronucleus from micronuclear DNA in ciliates by extensive programmed DNA breakage, rearrangements, and amplification is another example [38]. All these facts suggest the existence of physiological cellular mechanisms for limited or extensive DNA breakage in different cell types at different stages of development in which different enzymes may operate, and the dramatic consequences for genomic integrity should they go awry.

The thousands of hot spots of spontaneous DSBs in human chromosomes described here also support this hypothesis. The RAFT procedure may provide a snapshot of preexisting transient physiological DSBs associated with different processes (e.g., replication, transcription, genome rearrangements, transpositions, formation of chromosomal structures, etc.) in different genomic regions. Our PCR experiments across several FT independently support this notion. Transient DSB hot spots could appear upon the removal of DNA supercoiling from nucleosomes or the removal of torsion at the borders of different types of higher-order chromosomal structures required for the correct regulation of gene expression. Thus, the mapping of DSBs, hot spots, and protected regions (such as the 50–250 kb domains we have described) could contribute to our understanding of higher-order chromosomal structures.

### FT and chromosomal fragile sites

Some mapped DSBs are in late replicated regions and in regions with a paucity of initiation sites, particular nucleotide content, or DNA secondary structures that may impair replication fork movement [9], [10]. Our data correlating known CFS in lymphocytes and FT densities in HEK293T cells in chr6 and in some other chromosomal regions (Figure 4) clearly support this view. These regions could correspond to areas of conserved synteny where replication initiation profiles are maintained [29], [39]. However, it is obviously not correct to make a direct comparison between epigenetically inherited CFS in different cell types, due to the fact that different chromosomal regions can be committed to fragility in different cell types by cell-type-specific replication programs [9], [40], [41]. We propose that one set of physiological DSBs at FT is transient and is repaired easily, but repair of another set is hampered due to the specificity of the replication timing in a particular cell type, by collision between replication and transcription complexes in some loci, or by nucleotide content, or by DNA or chromatin structures. The set of FT that is hardly repaired likely corresponds to cytologically detected chromosomal fragile sites. The data on increased fragility at FT that was observed under replication stalling induced by HU (Figure 8B) suggest the relation of the most part of FT to chromosomal fragile sites. It follows that most of the FT is conserved in different cell types, but breakage frequencies may vary (Figure 4 and Figure 8C). This view is confirmed by the fact that In *Drosophila*, FT often correspond to the dispersed I-HC regions that are characterized by late replication and chromosomal breaks, and are involved in ectopic pairing [22]. The RAFT approach described here could be used to study scattered islands of putative heterochromatin in human chromosomes that are probably associated with fragile sites [26].

### What enzyme produces the DNA breaks at FT?

We do not know what enzyme(s) is responsible for the chromosomal fragmentation. Topoisomerase I is required for CFS breaks, suggesting that polymerase–helicase uncoupling could be involved in the breakage [42]. In *Drosophila*, FT sites often correspond to the binding sites of 19–24 nt RNAs and Argonaute proteins [22] suggesting the involvement of RNA-related mechanisms in the formation of DSB hot spots. The regulation of the elimination of macronuclear DNA by small non-coding RNAs during the formation of the ciliate micronucleus [38] is consistent with this. Whole-genome comparison of the parental macronucleus with the micronucleus led to conclusion that small RNAs are used to identify eliminated DNA sequences. However, small RNAs are also thought to act as guide molecules, directing recruitment of protein complexes to DSBs to facilitate repair [43]. Topo II is a good known candidate to produce extensive DSBs [44], and the consensus sequence for this enzyme has been described [45]. We used both the EMBOSS alignment tool (http://www.ebi.ac.uk/Tools/emboss/align/index.html) and WebLogo (http://weblogo.berkeley.edu/logo.cgi) to find either the 12-nt topo II consensus sequence or to define any consensus sequence in a number of FT, using the sequences around the cut sites, but we did not observe any consensus. This may indicate that the topo II site (5′ ANCNT[A/G]T.NN[G/C]N[A/G] 3′) is actually more variable or that this enzyme is not involved in production of the majority of the mapped DSBs.

### Coordinated expression in forum domains

Initially, gene clusters exhibiting similar expression profiles shaping transcriptional territories were described in *Drosophila* [24], [25]. In *Drosophila*, about 20% of the forum domains were transcriptionally active and contained co-expressed genes, whereas the majority of domains contained silent gene clusters [22]. Analysis of epigenetic modifications in human chromosomes revealed spatially coherent combinations of chromatin marks and large-scale repressed and inactive domains [30], [46]. These data are consistent with our analysis of transcription in forum domains. The data shown in Figure 6A and in Figures S13 and S14 suggests that the same silenced forum domains could be present in different cell types. The data shown in Table S3 confirm this conclusion. In three more cell lines (IMR90 cell line, originated from fetal lung fibroblasts, K-562 cell line, originated form pleural cells, and embryonic stem cells) we detected that from 74 to 92% of forum domains in different chromosomes have low expression or are silent. The presence of hot spots of DSBs at the borders of silenced or actively expressed gene clusters possessing different chromatin marks is consistent with the view that DSBs are involved in reducing topological stress imposed by long regions of uniform chromatin states. The data presented in Text S1 describing the usage of the circular permutation approach for genome-wide analysis of expression levels of genes in the same forum domain strongly argues in favor of coordinated expression of genes in forum domains.

We speculate that small-RNA-directed DNA elimination in ciliates may lead to the selection of actively transcribed DNA domains that correspond to actively expressed forum domains, which accumulate in the macronucleus. In this way, widespread DNA breakage would lead to the sequestration of the active portion of the genome.

### Binding of master regulators at the borders of forum domains

The specific binding of PARP1 and HNRNPA2B1 proteins at FT sequences is very intriguing. PARP1 is a known regulator of chromatin structure and transcription [47], [48]. Our RAFT probes also bound to DNA-PK, K80, and K70, but this is a non-specific DNA-end-binding activity [49], [50] that was eliminated in the presence of an excess of the prokaryotic PCR-amplified DNA used as a source of free DNA ends. The binding of PARP1 to FT may hint at the nature of the coordinated gene expression we observed within forum domains. PARP1 was recently shown to mediate the inheritance of silent chromatin state epigenetic signatures via non-coding RNA [51]–[53], consistent with our findings.

HNRNPA2B1 belongs to the A/B subfamily of ubiquitously expressed heterogeneous nuclear ribonucleoproteins that bind to pre-mRNAs and are involved in the splicing and nucleocytoplasmic transport of mRNAs [54]. The protein is known to be involved in cell proliferation and in tumor invasion [55]. It is possible that the binding of this protein at FT influences coordinated transcription within forum domains. We believe that the detection of HNRNPA2B1 binding at thousands of FT scattered throughout the human genome will promote the further study of its mechanistic role in the functioning of chromosomal domains. PARP1 and HNRNPA2B1 have not previously been considered to function together. However, recently it was described that HNRNPA2B1 is an H2AX interacting protein [56].

### A novel type of regulation

Our data suggest the presence of a novel type of gene regulation: a coordinated silencing or transcription of gene clusters within large chromosomal domains that are protected from fragmentation, delimited by non-randomly dispersed DSB hot spots and the binding sites of PARP1 and HNRNPA2B1. They highlight the relationship between human chromosomal fragile sites, chromosomal organization, and gene regulation, which may prove to be important for cancer genomics and gene therapy. The data strongly suggest a mechanistic link between DSBs, transcription patterns, and genomic instability. This outcome is in agreement with the recent conclusion that mapping of DSBs provides a novel tool to analyze genome architecture and that DSBs are distributed much more uniformly than was previously believed [57], [58]. When this paper was in revision, data describing the important role of PARP1 in somatic cell reprogramming were published [59]. The data clearly support our conclusion about the important role of PARP1in regulation of expression in chromosomal domains. Our current research aims to address the details of this type of regulation within individual forum domains.

## Materials and Methods

### RAFT preparation

The RAFT procedure was performed as described [22]. About 1.5 µg of isolated DNA was treated with the Klenow fragment of *E. coli* DNA polymerase I and then ligated with a molar excess of double-stranded biotinylated oligonucleotide (see details in Text S1). The DNA was then digested with Sau3A to shorten the forum domain to the termini attached to the ligated oligonucleotide. The selection of FT was performed using SA-PMP (Promega, Madison, WI, USA) according to the manufacturer's recommendations. After extensive washing, the FT DNA preparation was eluted from the SA-PMP. The FT were then ligated with a 100-fold molar excess of double-stranded Sau3A adaptor. The final DNA samples were amplified by PCR.

### FISH

Total DNA isolated from HEK 293T cells and RAFT preparations (4 µg each) was labeled with Alexa Fluor 5 and Alexa Fluor 3, respectively, using a BioPrime total genomic labeling system (Invitrogen) according to the manufacturer's recommendations. G-banding was performed prior to FISH by use of a standard procedure [60]. Metaphases were photographed, and slides were de-stained in methanol and fixed with 0.5% formaldehyde. FISH was performed using a standard protocol [61], [62].

### Nuclear protein extracts

Nuclear proteins from HEK293T cells were isolated as described [63]. The final protein concentration, measured using a NanoDrop 2000 Spectrophotometer, was about 1–3 µg/µL.

### Isolation of proteins binding to RAFT preparations

Initially, 150 µL of nuclear protein extract (150–300 µg of protein) was pre-exhausted for 10 min at 10°C in 400 µL of solution containing 20 mM HEPES, pH 7.6, 4% Ficoll, 5 mM MgCl_2_, 0.2 mM EDTA, 1 mM DTT, and 30 µg of poly[d(I)/d(C)]. After pre-incubation, 0.4 µg of biotinylated RAFT preparation was added, and incubation at 20°C was carried out for 1 h with gentle mixing every 10 min. The bound proteins were selected on SA-PMP according to the manufacturer's recommendations.

### Chromatin immunoprecipitation

The HEK 293T cell suspension was treated with 1% formaldehyde at 20°C for 10 min. The nuclei were washed and lysed, and chromatin was sheared to an average length of 600 bp by sonication. X-ChIP was carried out using the OneDay ChIP kit (Diagenode, Liege, Belgium), with 4 mg of antibodies against PARP1 (ActiveMotif, Carlsbad, CA, USA) or HNRNPA2B1 (Sigma Aldrich, St. Louis, MO, USA). The negative control was DNA precipitated using 4 mg of non-specific IgG from rabbit serum. The PCR primers corresponding to FT and non-FT regions are listed in Tables S1 and S2.

### Computer treatment of data

Raw data in SFF format were obtained using a 454 Roche GS FLX Life Sciences pyrosequencing machine. Data were then decoded to FASTQ/FASTA format using PyroBayes (http://bioinformatics.bc.edu/marthlab/PyroBayes). Elimination of primers sequences was performed by Perl script using BioPerl as interface to FASTA, with the assumption that a primer should be at either end of a read, but not further than 5 bp from it. All sequences shorter than 18 bp were removed from the dataset. The final mapping was performed using BWA (http://bio-bwa.sourceforge.net) and Samtools (http://samtools.sourceforge.net) with the *Homo sapiens* masked genome (assembly GRCh37p5/hg19) as the database (taken in the form of MFA files from ftp://ftp.ncbi.nih.gov/genomes/H_sapiens/Assembled_chromosomes/seq). The saturation curve indicating that practically all FT were defined is shown in Figure S15.

### Statistical analysis

All domain–gene comparisons and statistical evaluations were performed with each gene's database from the same GRCh37p5 genome build (taken in the form of GBS files from ftp://ftp.ncbi.nih.gov/genomes/H_sapiens/Assembled_chromosomes/gbs) by the Perl script with the BPLITE module as the interface for GBK/GBS files. Monte-Carlo simulations were performed using the following procedure. First, our investigations revealed that Perl's built-in random module (Math::Random) generates poor quality pseudorandom numbers. Therefore, we used the Mersenne Twister pseudorandom generator [64], an algorithm designed for rapid generation of very high-quality pseudorandom numbers and created to rectify many of the flaws found in previously developed algorithms). For further simulations, we created 10,000 datasets per chromosome with randomly created breaks within the same length limits and numbers as found in forum domains. Further statistical evaluations were performed using Perl script within the interface Statistics::R to R project (http://www.r-project.org). Because the standard interface sometimes cannot process the R output correctly, we reworked the processing procedure to meet our own needs. The details are described in Text S1.

### Accession number

The mapping result was deposited into the GEO database with the accession ID GSE35065 (http://www.ncbi.nlm.nih.gov/geo/query/acc.cgi?acc=GSE35065). The reads are presented in .gff and .wig files, which are divided by chromosomes for convenience. The data in the .gff and .wig files are the same—only the format differs.

## Supporting Information

Figure S1 Overviews of chr1, chr4, chr5, chr8, and chr9–chr12. Integrated Genome Browser (Affymetrix) was used. The FT barcode is shown in red.
(TIF)

Figure S2 Overviews of chr13, chr14, chr15, chr17, chr18, chr19, chr20, chr21, and chr22. Integrated Genome Browser (Affymetrix) was used. The FT barcode is shown in red.
(TIF)

Figure S3 Overviews of chr2, chr6, chr7, and chrX. Integrated Genome Browser (Affymetrix) was used. The FT barcode is shown in red. The length and numbers of reads are shown above the barcode in chrX. The frequently and less frequently observed CFS detected in leukocytes are shown in red and in blue, respectively.
(TIF)

Figure S4 Z-DNA region and palindromes inside the 1090 bp sequence that possesses the mapped FT in the 3′ exon of the *WWOX* gene. This sequence escapes cloning in *E. coli* cells. The Z-DNA region (highlighted in yellow) was detected using ZHunt Online software (http://gac-web.cgrb.oregonstate.edu/zDNA/index). The region corresponding to FT reads is shown in blue (the *Sau* site is indicated in red). The underlined portion is a shorter amplified DNA fragment that also escapes cloning. Folding of the 1090 bp fragment was performed using the UNAFold Web Server http://mfold.rna.albany.edu/?q=mfold/DNA-Folding-Form).
(PDF)

Figure S5 Quantitative real-time PCR across the FT in the 3′ exon in the *WWOX* gene. The results of four independent experiments are shown. Different forum domains (Forum) and control DNA (Intact) preparations, isolated as described in Text S1, were used.
(PDF)

Figure S6 Expression levels inside forum domains in chr4, chr5, and chr6. The data for expression in HEK293T cells (wgEncodeEH002692_2) were used. The median values of transcription levels in coding regions (representing exon array signals) within a particular forum domain were used, and the result was plotted according to the position of the domain in its chromosome. The arrows indicate the position of the average expression level of forum domains in a particular chromosome. The value to the right of the arrow indicates the portion of forum domains in a chromosome that is more highly expressed.
(PDF)

Figure S7 Expression levels inside forum domains in chr7, chr8, and chr9. The data for expression in HEK293T cells (wgEncodeEH002692_2) were used. The median values of transcription levels in coding regions (representing exon array signals) within a particular forum domain were used, and the result was plotted according to the position of the domain in its chromosome. The arrows indicate the position of the average expression level of forum domains in a particular chromosome. The value to the right of the arrow indicates the portion of forum domains in a chromosome that is more highly expressed.
(PDF)

Figure S8 Expression levels inside forum domains in chr10, chr11, and chr12. The data for expression in HEK293T cells (wgEncodeEH002692_2) were used. The median values of transcription levels in coding regions (representing exon array signals) within a particular forum domain were used, and the result was plotted according to the position of the domain in its chromosome. The arrows indicate the position of the average expression level of forum domains in a particular chromosome. The value to the right of the arrow indicates the portion of forum domains in a chromosome that is more highly expressed.
(PDF)

Figure S9 Expression levels inside forum domains in chr13, chr14, and chr15. The data for expression in HEK293T cells (wgEncodeEH002692_2) were used. The median values of transcription levels in coding regions (representing exon array signals) within a particular forum domain were used, and the result was plotted according to the position of the domain in its chromosome. The arrows indicate the position of the average expression level of forum domains in a particular chromosome. The value to the right of the arrow indicates the portion of forum domains in a chromosome that is more highly expressed.
(PDF)

Figure S10 Expression levels inside forum domains in chr16, chr17, and chr18. The data for expression in HEK293T cells (wgEncodeEH002692_2) were used. The median values of transcription levels in coding regions (representing exon array signals) within a particular forum domain were used, and the result was plotted according to the position of the domain in its chromosome. The arrows indicate the position of the average expression level of forum domains in a particular chromosome. The value to the right of the arrow indicates the portion of forum domains in a chromosome that is more highly expressed.
(PDF)

Figure S11 Expression levels inside forum domains in chr19, chr20, and chr21. The data for expression in HEK293T cells (wgEncodeEH002692_2) were used. The median values of transcription levels in coding regions (representing exon array signals) within a particular forum domain were used, and the result was plotted according to the position of the domain in its chromosome. The arrows indicate the position of the average expression level of forum domains in a particular chromosome. The value to the right of the arrow indicates the portion of forum domains in a chromosome that is more highly expressed.
(PDF)

Figure S12 Expression levels inside forum domains in chr22 and chrX (A) and the average expression levels per forum domain in different chromosomes (B). The data for expression in HEK293T cells (wgEncodeEH002692_2) were used. The median values of transcription levels in coding regions (representing exon array signals) within a particular forum domain were used, and the result was plotted according to the position of the domain in its chromosome. The arrows indicate the position of the average expression level of forum domains in a particular chromosome. The value to the right of the arrow indicates the portion of forum domains in a chromosome that is more highly expressed.
(PDF)

Figure S13 Coordinated expression inside the 498 kb forum domain that possesses the *HOX*A gene cluster. The UCSC Genome Browser on Human Feb. 2009 (GRCh37/hg19) Assembly was used. UCSC genes, Human mRNAs from GenBank, the H3K37Ac mark from Encode, chromatin state segmentation by HMM from Encode/Broad, and some histone modifications by ChIP-Seq from Encode are indicated. The “RNA-seq” lane corresponds to expression of mRNAs in IMR90 cells (GEO accession number GSM438363). Forum domain containing actively transcribed genes are indicated by the red bracket. “HEK293T” lanes correspond to the expression of mRNA in HEK293T cells (microarray data using Affymetrix Human Exon 1.0 ST expression arrays, wgEncodeEH002692_2).
(TIF)

Figure S14 Coordinated expression inside the 374 kb forum domain that possesses the *HOXC* gene cluster (A) and inside the 673 kb forum domain that possesses the *HOXD* gene cluster (B). The UCSC Genome Browser on Human Feb. 2009 (GRCh37/hg19) Assembly was used. UCSC genes, Human mRNAs from GenBank, the H3K37Ac mark from Encode, chromatin state segmentation by HMM from Encode/Broad, and some histone modifications by ChIP-Seq from Encode are indicated. The “RNA-seq” lane corresponds to expression of mRNAs in IMR90 cells (GEO accession number GSM438363). Forum domains containing actively transcribed genes are indicated by the red bracket. “Domains” lanes indicate the forum domains containing the silent or weakly expressed genes (blue brackets). “HEK293T” lanes correspond to the expression of mRNA in HEK293T cells (microarray data using Affymetrix Human Exon 1.0 ST expression arrays, wgEncodeEH002692_2).
(TIF)

Figure S15 Saturation curve. % of FT plotted against decreasing % of reads reveals a plateau in the range between 90 and 100% of reads. The step was equal to 1% of reads in the range from 100% towards 70% and 5% down to 10% of reads. The curve indicates that practically all FT, corresponding to hot spots of DSBs, were defined.
(PDF)

Table S1 Primers used in quantitative PCR experiments.
(DOC)

Table S2 Primers used in the ChIP experiments shown in Figure 9C.
(DOC)

Table S3 Percent of low expressing or silent forum domains in four cell lines. Names of cell lines and their corresponding accession numbers are indicated. The conversion of data for the second and third columns from hg18 to hg19 coordinates was performed using the LiftOver program (http://hgdownload.cse.ucsc.edu/admin/exe). The median values of transcription levels in coding regions (representing exon array signals) within a particular forum domain were used. The average expression level of forum domains in a particular chromosome was determined as a sum of expression data per chromosome divided into domains numbers. The values in the last four columns correspond to % of low expressing (below the average expression level in a particular chromosome) or silenced forum domains in a corresponding chromosome. Most domains in different cell lines are silent or expressed at very low levels.
(DOC)

Text S1 Extended Materials and Methods.
(DOC)
