# Supplementary figures and images for: DNA Double-Strand Breaks Coupled with PARP1 and HNRNPA2B1 Binding Sites Flank Coordinately Expressed Domains in Human Chromosomes
Source: PLoS Genet. 2013 Apr 4;9(4):e1003429. doi: 10.1371/journal.pgen.1003429 (PMC3616924; doi:10.1371/journal.pgen.1003429)

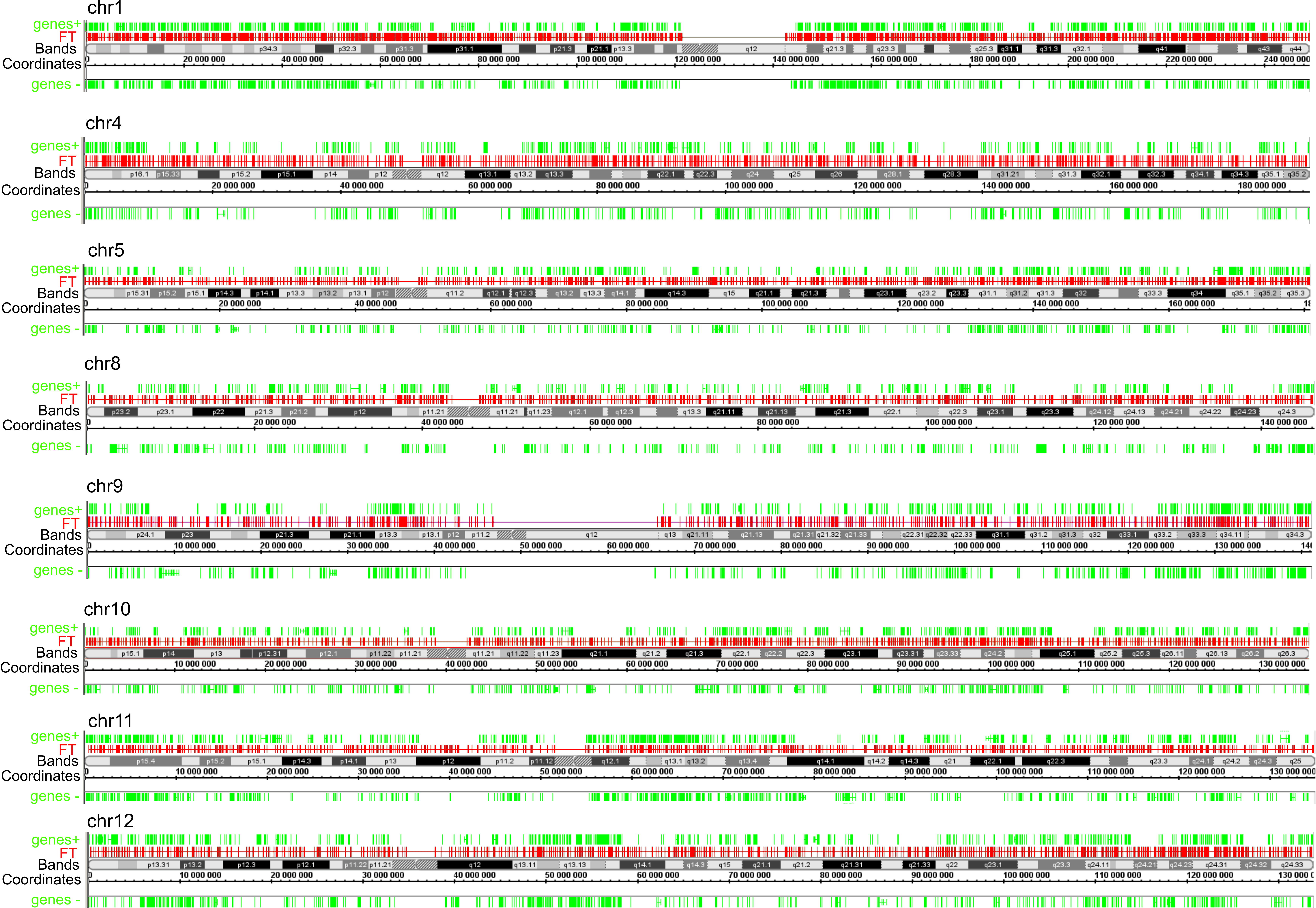

Supplement: Figure S1 — Overviews of chr1, chr4, chr5, chr8, and chr9–chr12. Integrated Genome Browser (Affymetrix) was used. The FT barcode is shown in red. (TIF) [file pgen.1003429.s001.tif]

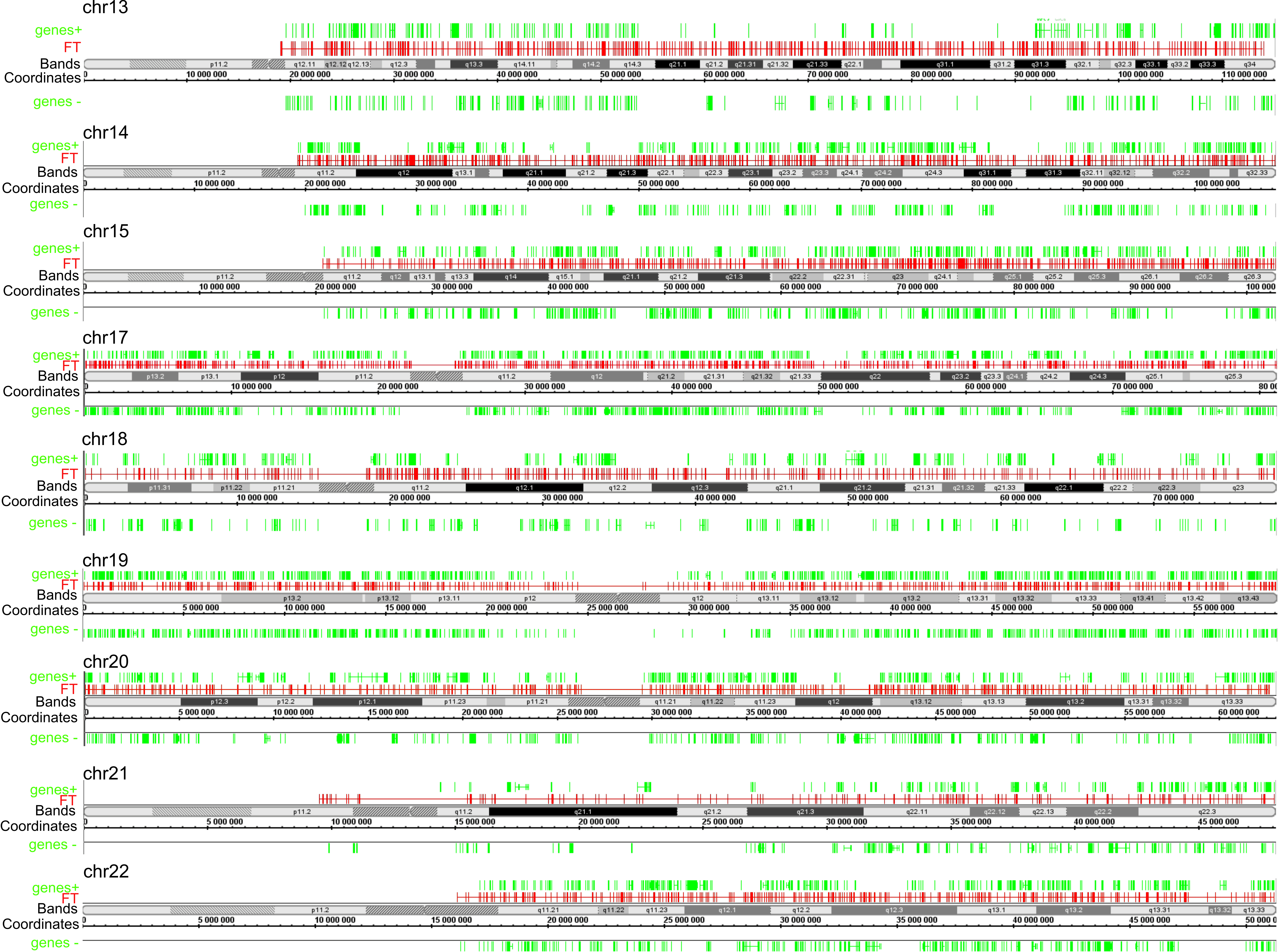

Supplement: Figure S2 — Overviews of chr13, chr14, chr15, chr17, chr18, chr19, chr20, chr21, and chr22. Integrated Genome Browser (Affymetrix) was used. The FT barcode is shown in red. (TIF) [file pgen.1003429.s002.tif]

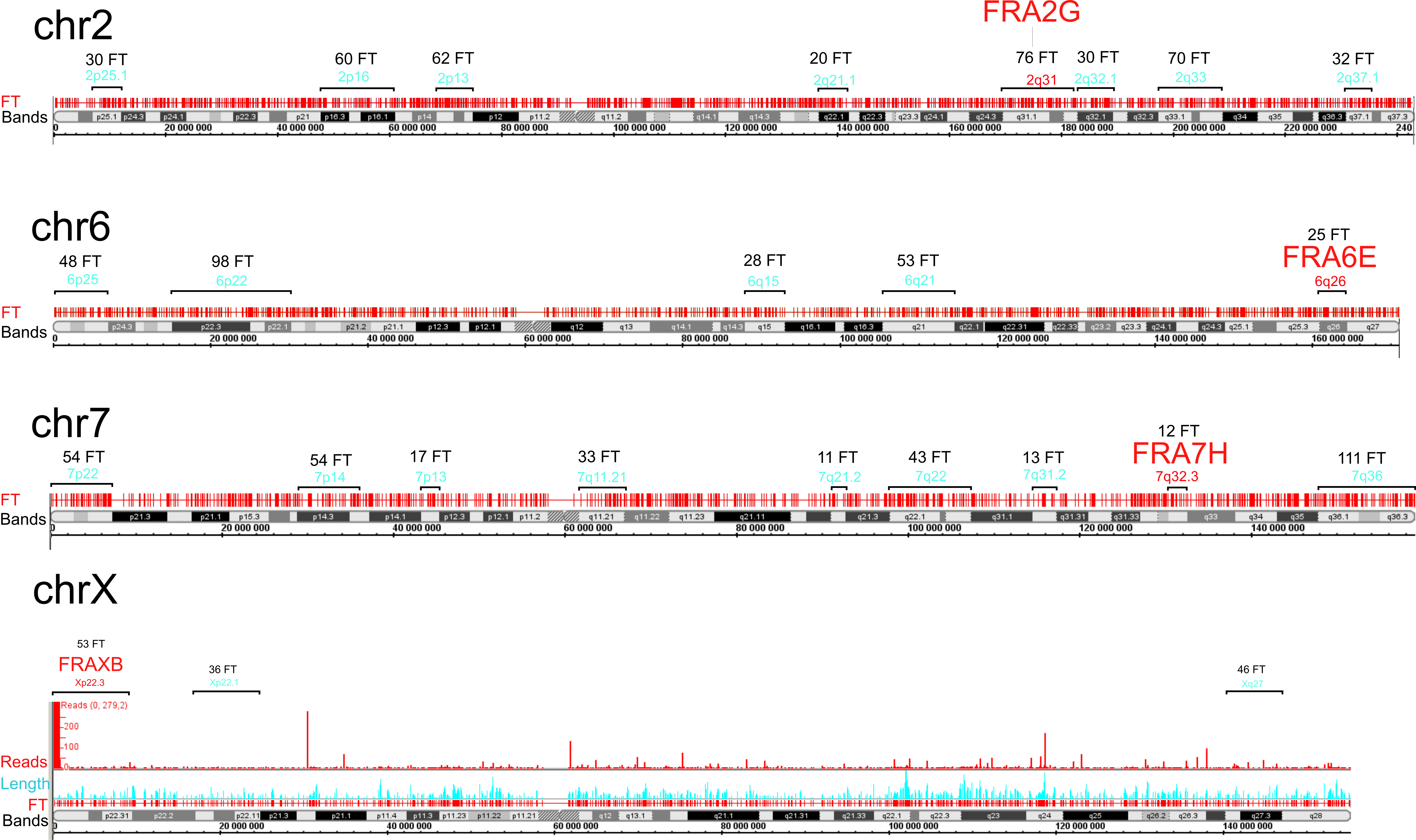

Supplement: Figure S3 — Overviews of chr2, chr6, chr7, and chrX. Integrated Genome Browser (Affymetrix) was used. The FT barcode is shown in red. The length and numbers of reads are shown above the barcode in chrX. The frequently and less frequently observed CFS detected in leukocytes are shown in red and in blue, respectively. (TIF) [file pgen.1003429.s003.tif]

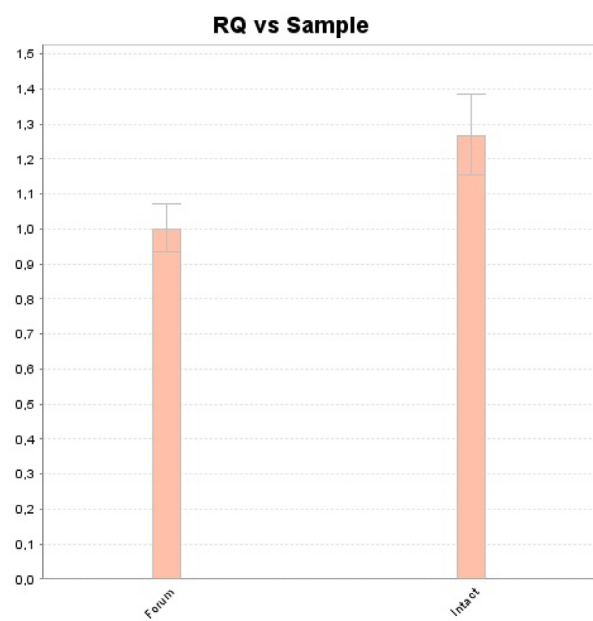

Supplement: Figure S5 — Quantitative real-time PCR across the FT in the 3′ exon in the WWOX gene. The results of four independent experiments are shown. Different forum domains (Forum) and control DNA (Intact) preparations, isolated as described in Text S1, were used. (PDF) [file pgen.1003429.s005.pdf]

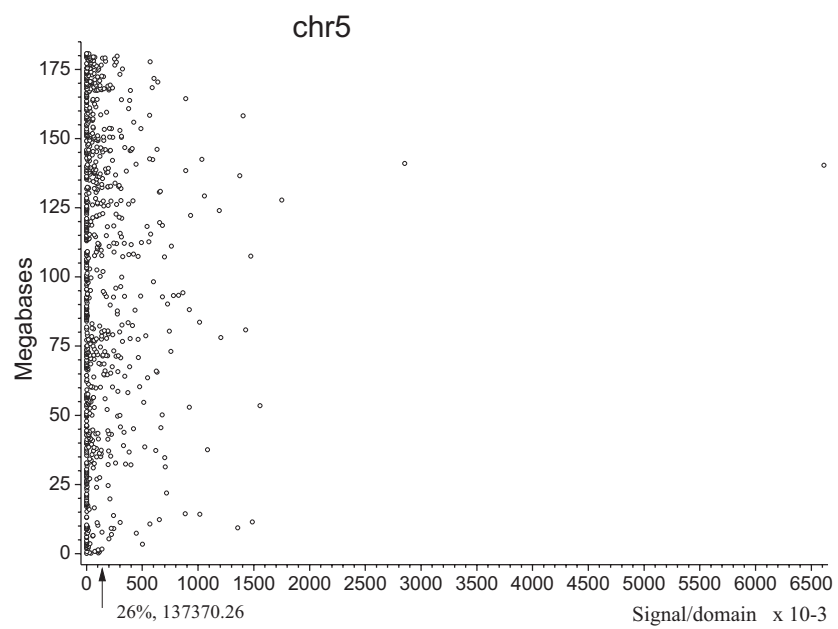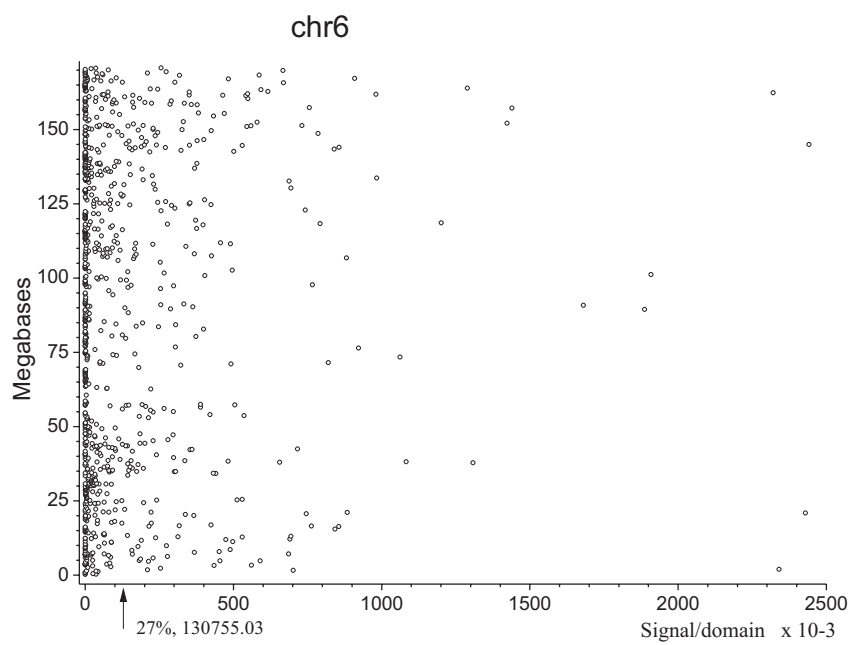

Supplement: Figure S6 — Expression levels inside forum domains in chr4, chr5, and chr6. The data for expression in HEK293T cells (wgEncodeEH002692_2) were used. The median values of transcription levels in coding regions (representing exon array signals) within a particular forum domain were used, and the result was plotted according to the position of the domain in its chromosome. The arrows indicate the position of the average expression level of forum domains in a particular chromosome. The value to the right of the arrow indicates the portion of forum domains in a chromosome that is more highly expressed. (PDF) [file pgen.1003429.s006.pdf]

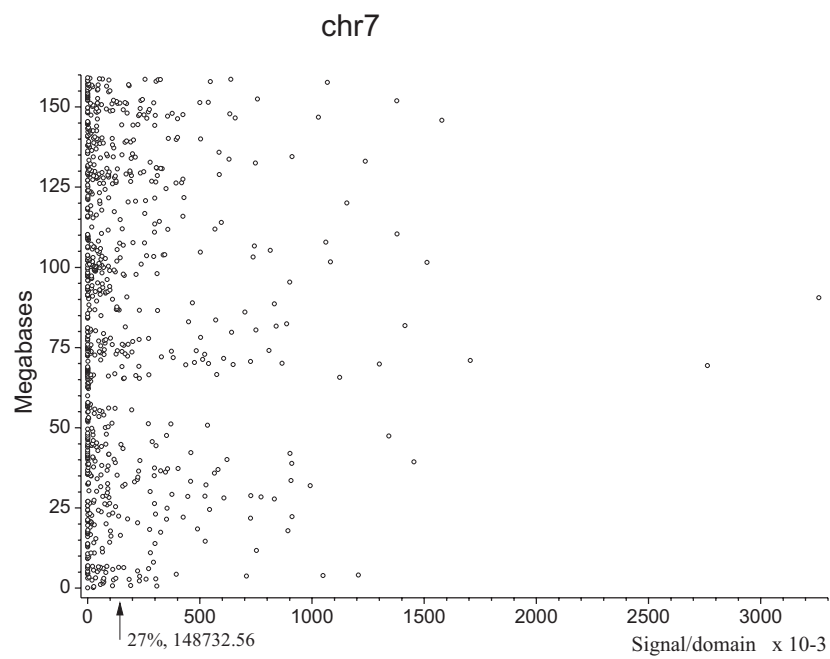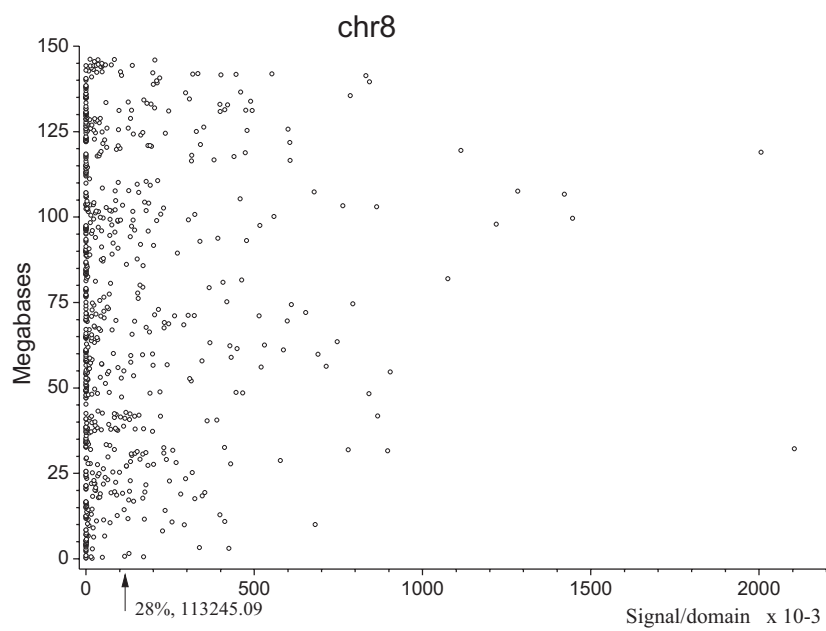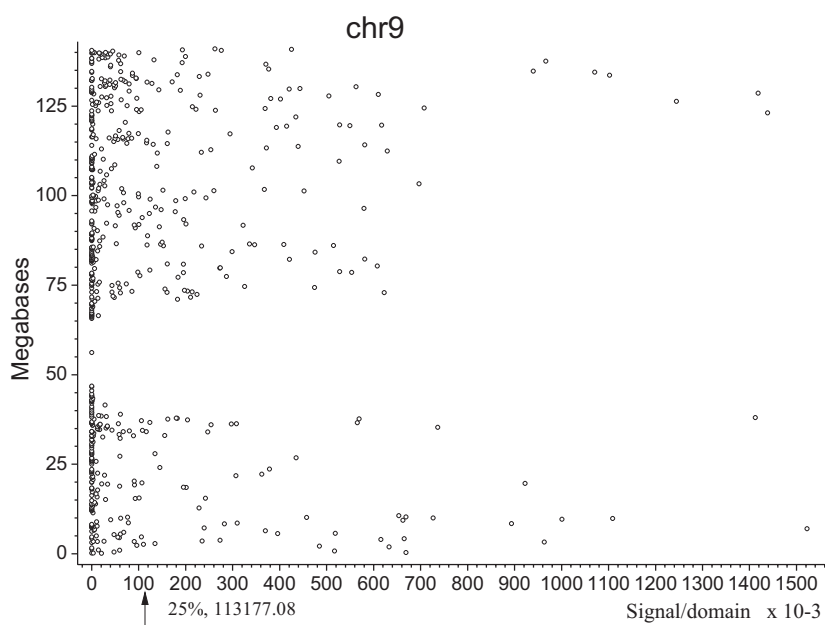

Supplement: Figure S7 — Expression levels inside forum domains in chr7, chr8, and chr9. The data for expression in HEK293T cells (wgEncodeEH002692_2) were used. The median values of transcription levels in coding regions (representing exon array signals) within a particular forum domain were used, and the result was plotted according to the position of the domain in its chromosome. The arrows indicate the position of the average expression level of forum domains in a particular chromosome. The value to the right of the arrow indicates the portion of forum domains in a chromosome that is more highly expressed. (PDF) [file pgen.1003429.s007.pdf]

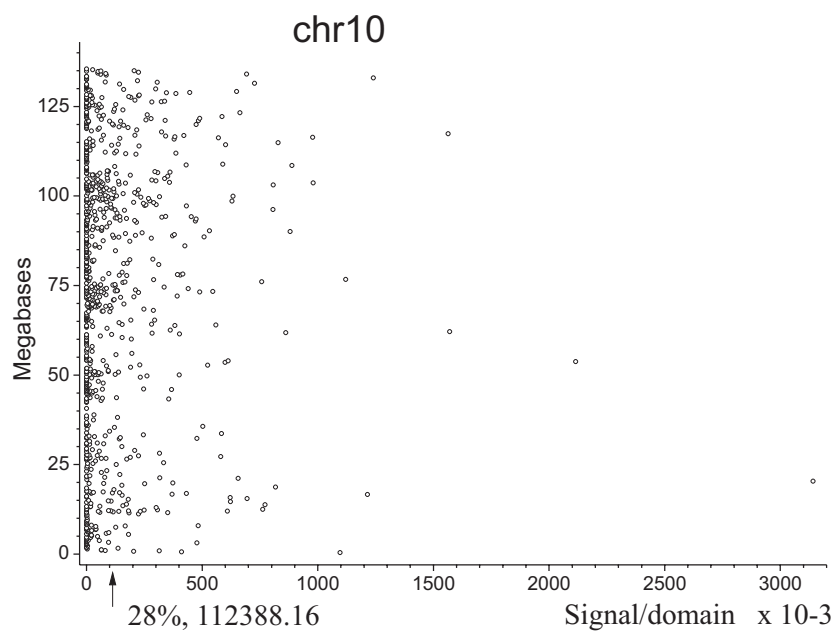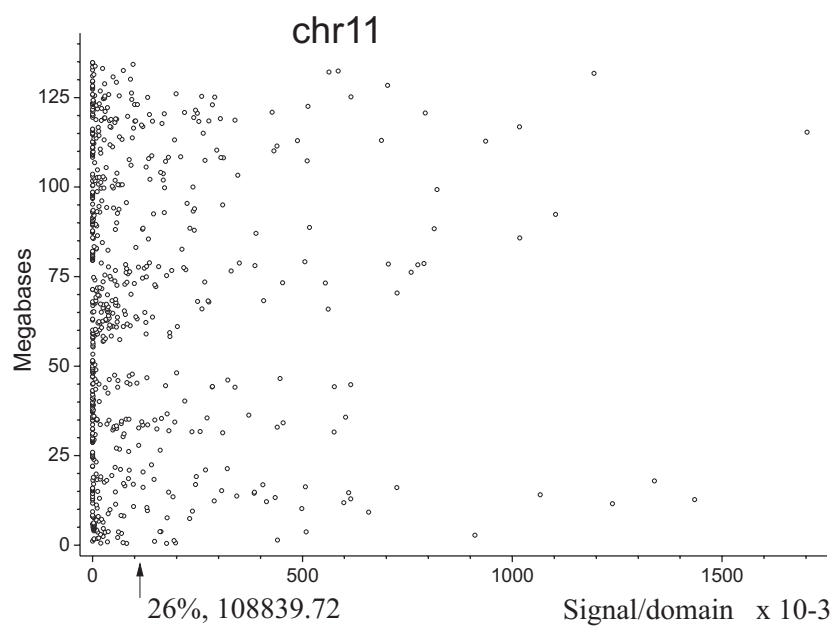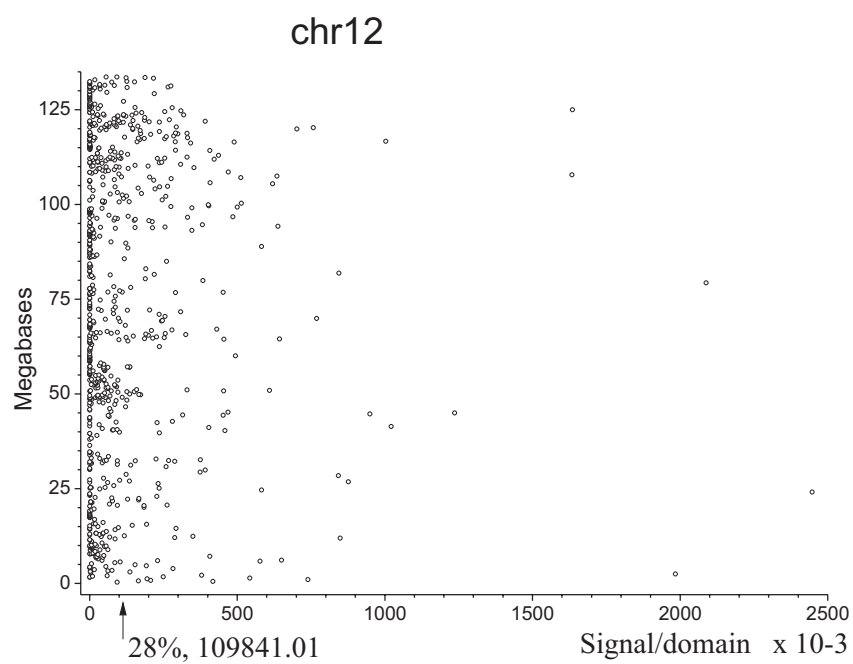

Supplement: Figure S8 — Expression levels inside forum domains in chr10, chr11, and chr12. The data for expression in HEK293T cells (wgEncodeEH002692_2) were used. The median values of transcription levels in coding regions (representing exon array signals) within a particular forum domain were used, and the result was plotted according to the position of the domain in its chromosome. The arrows indicate the position of the average expression level of forum domains in a particular chromosome. The value to the right of the arrow indicates the portion of forum domains in a chromosome that is more highly expressed. (PDF) [file pgen.1003429.s008.pdf]

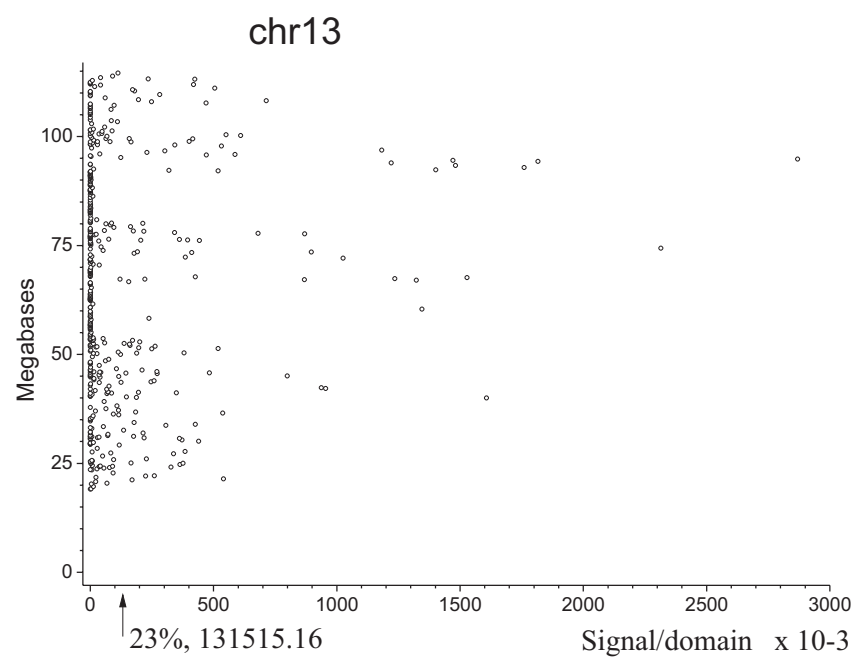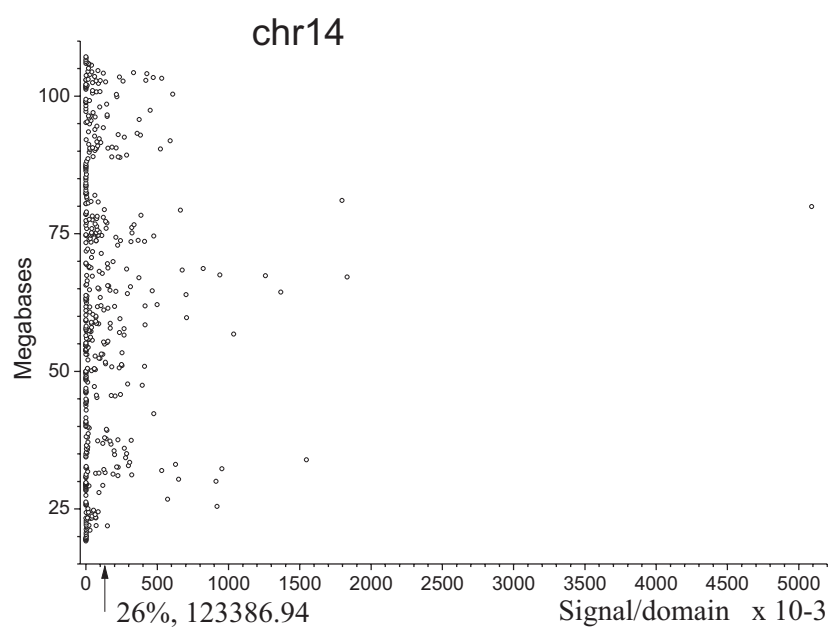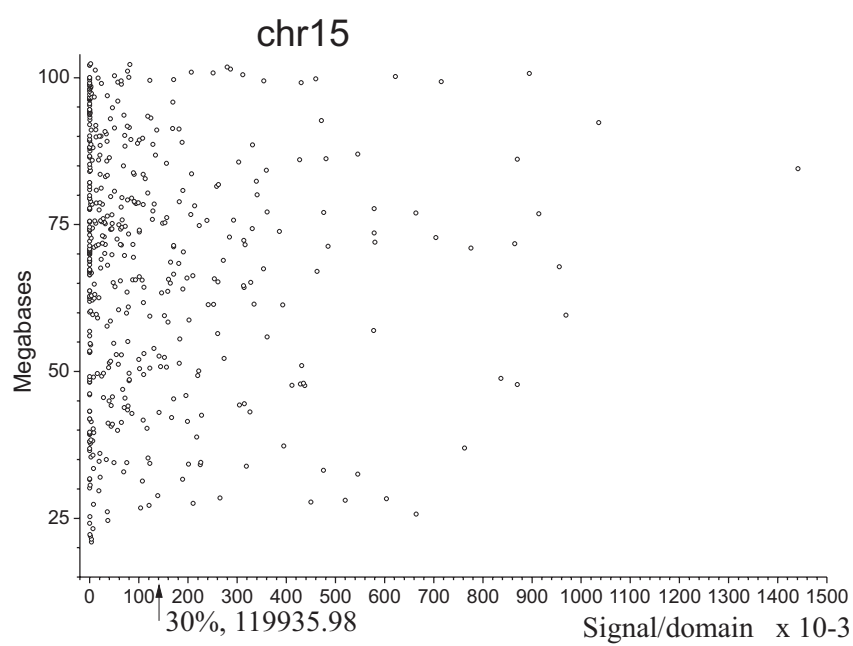

Supplement: Figure S9 — Expression levels inside forum domains in chr13, chr14, and chr15. The data for expression in HEK293T cells (wgEncodeEH002692_2) were used. The median values of transcription levels in coding regions (representing exon array signals) within a particular forum domain were used, and the result was plotted according to the position of the domain in its chromosome. The arrows indicate the position of the average expression level of forum domains in a particular chromosome. The value to the right of the arrow indicates the portion of forum domains in a chromosome that is more highly expressed. (PDF) [file pgen.1003429.s009.pdf]

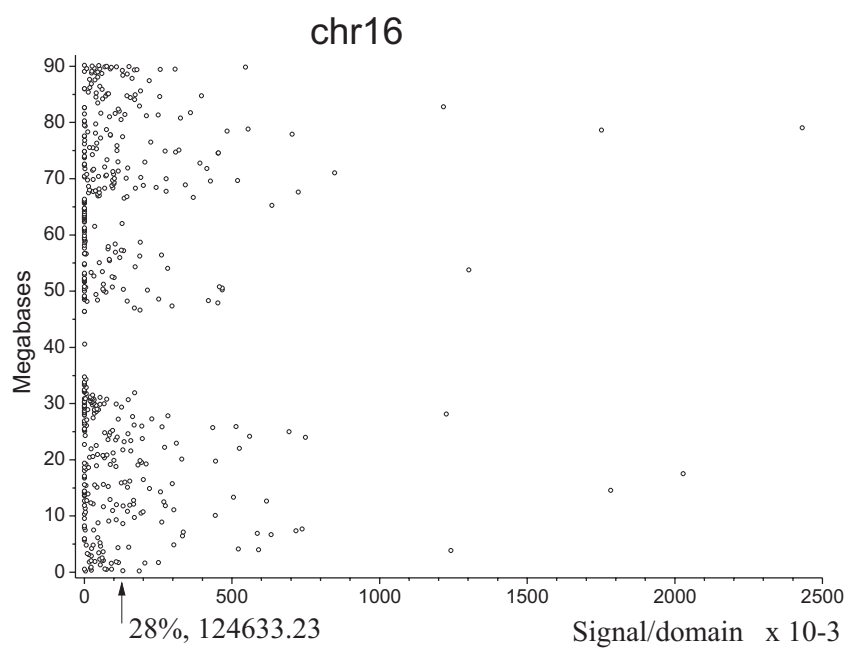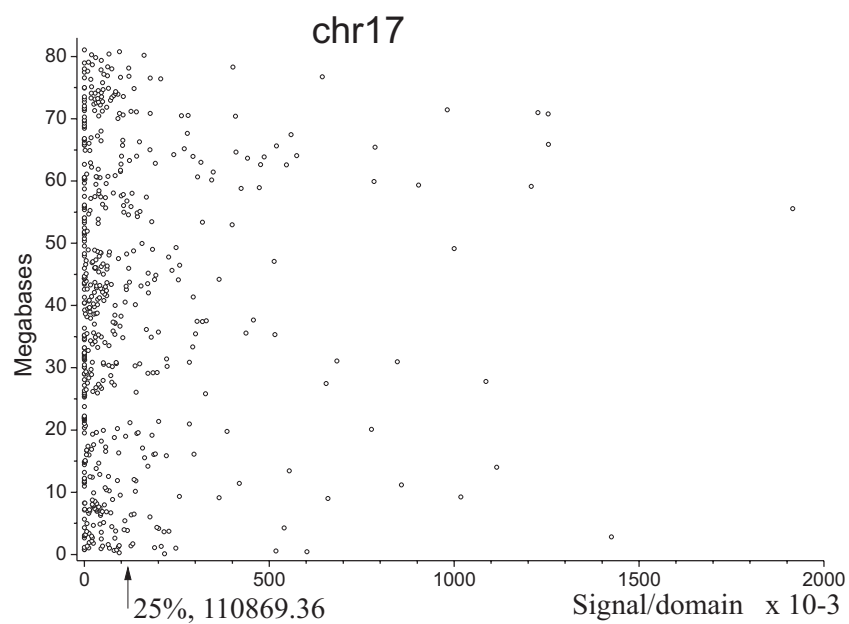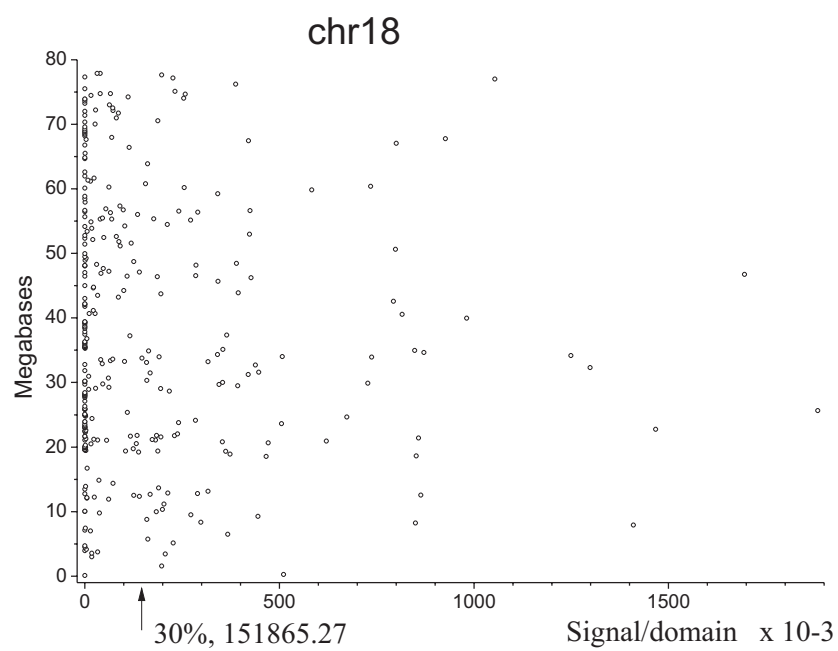

Supplement: Figure S10 — Expression levels inside forum domains in chr16, chr17, and chr18. The data for expression in HEK293T cells (wgEncodeEH002692_2) were used. The median values of transcription levels in coding regions (representing exon array signals) within a particular forum domain were used, and the result was plotted according to the position of the domain in its chromosome. The arrows indicate the position of the average expression level of forum domains in a particular chromosome. The value to the right of the arrow indicates the portion of forum domains in a chromosome that is more highly expressed. (PDF) [file pgen.1003429.s010.pdf]

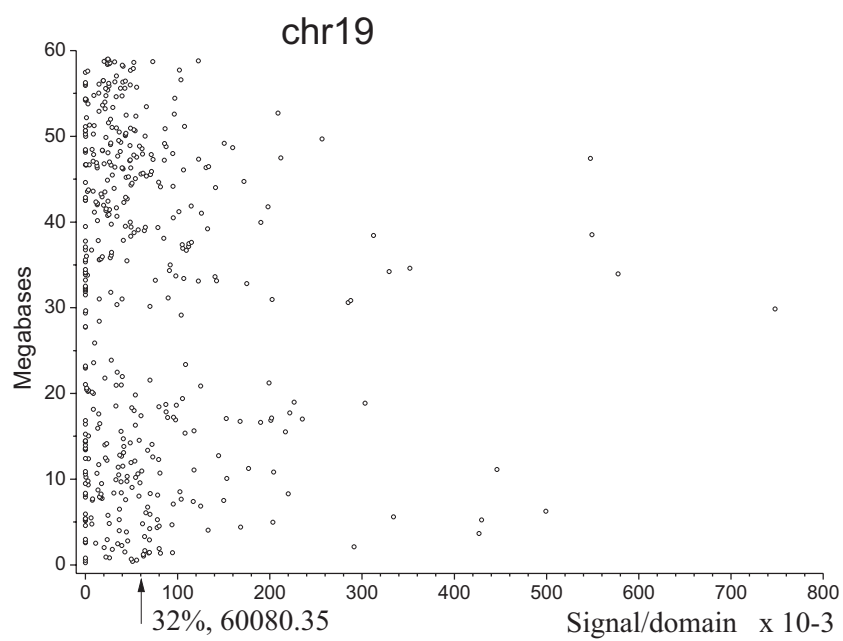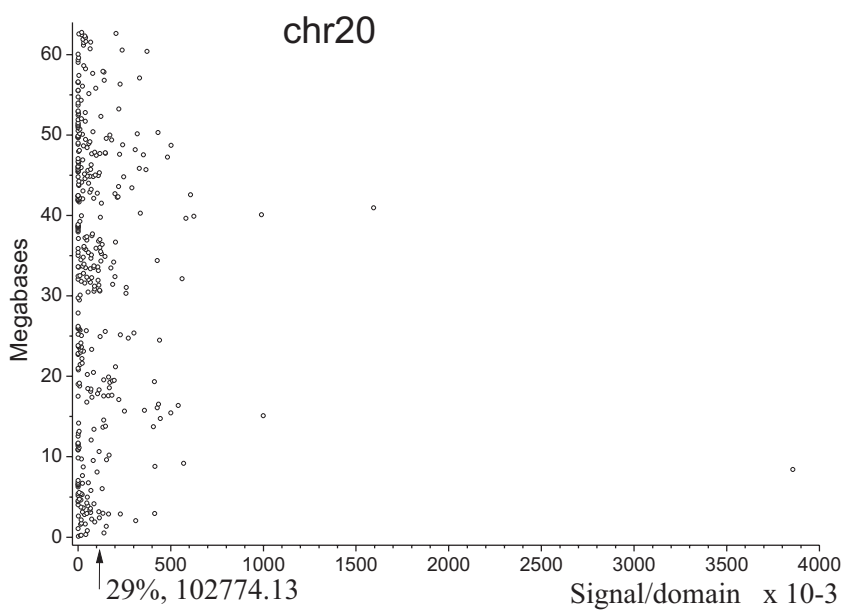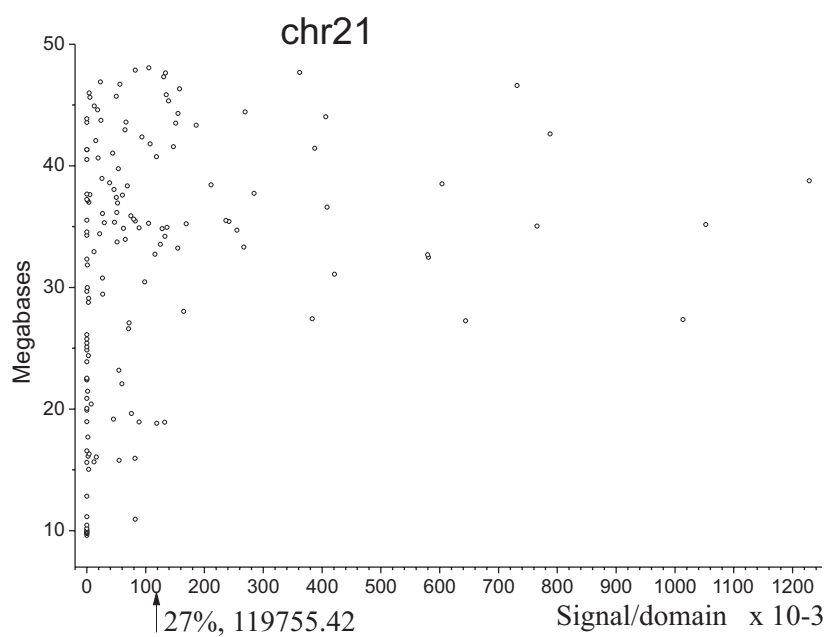

Supplement: Figure S11 — Expression levels inside forum domains in chr19, chr20, and chr21. The data for expression in HEK293T cells (wgEncodeEH002692_2) were used. The median values of transcription levels in coding regions (representing exon array signals) within a particular forum domain were used, and the result was plotted according to the position of the domain in its chromosome. The arrows indicate the position of the average expression level of forum domains in a particular chromosome. The value to the right of the arrow indicates the portion of forum domains in a chromosome that is more highly expressed. (PDF) [file pgen.1003429.s011.pdf]

A

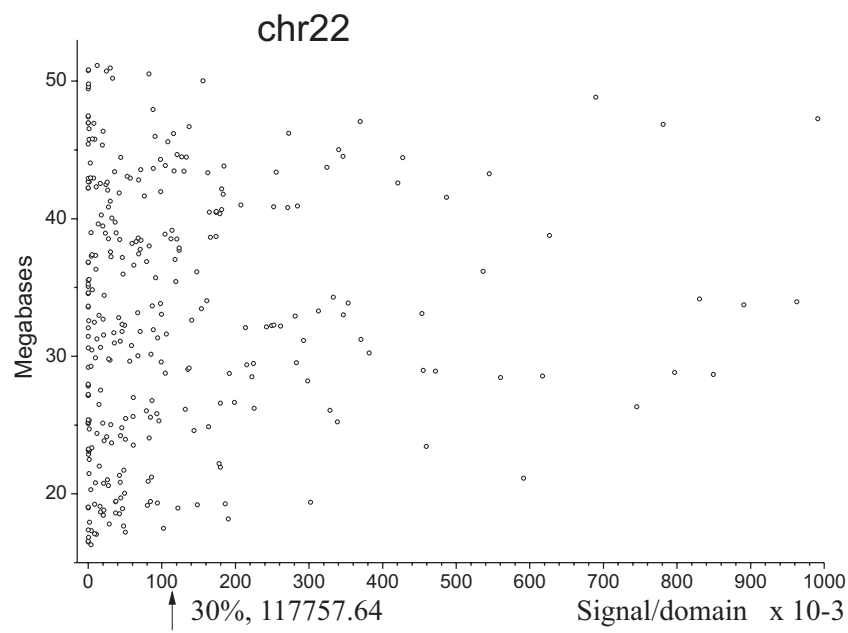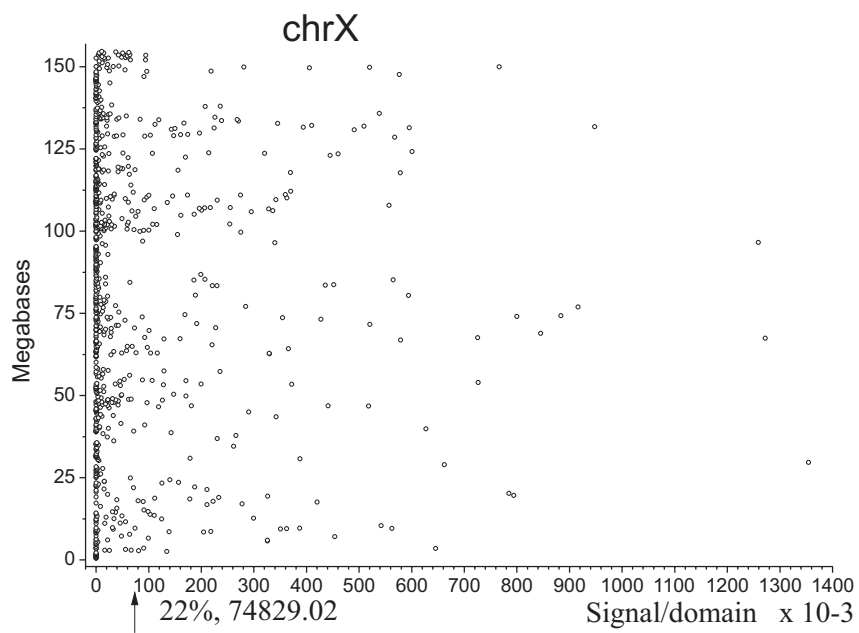

B

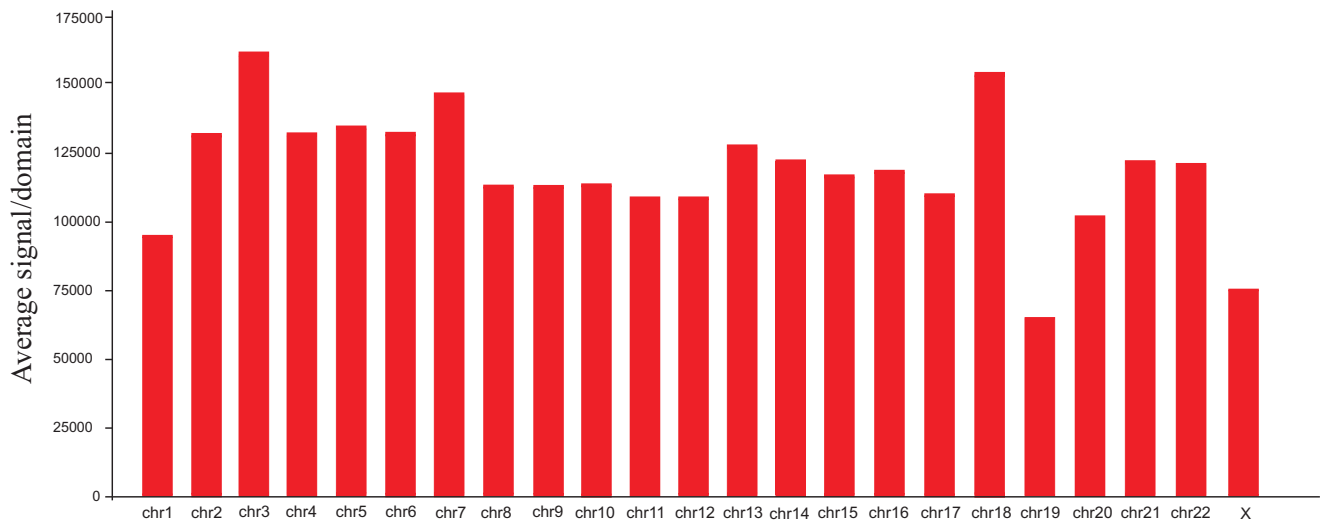

Supplement: Figure S12 — Expression levels inside forum domains in chr22 and chrX (A) and the average expression levels per forum domain in different chromosomes (B). The data for expression in HEK293T cells (wgEncodeEH002692_2) were used. The median values of transcription levels in coding regions (representing exon array signals) within a particular forum domain were used, and the result was plotted according to the position of the domain in its chromosome. The arrows indicate the position of the average expression level of forum domains in a particular chromosome. The value to the right of the arrow indicates the portion of forum domains in a chromosome that is more highly expressed. (PDF) [file pgen.1003429.s012.pdf]

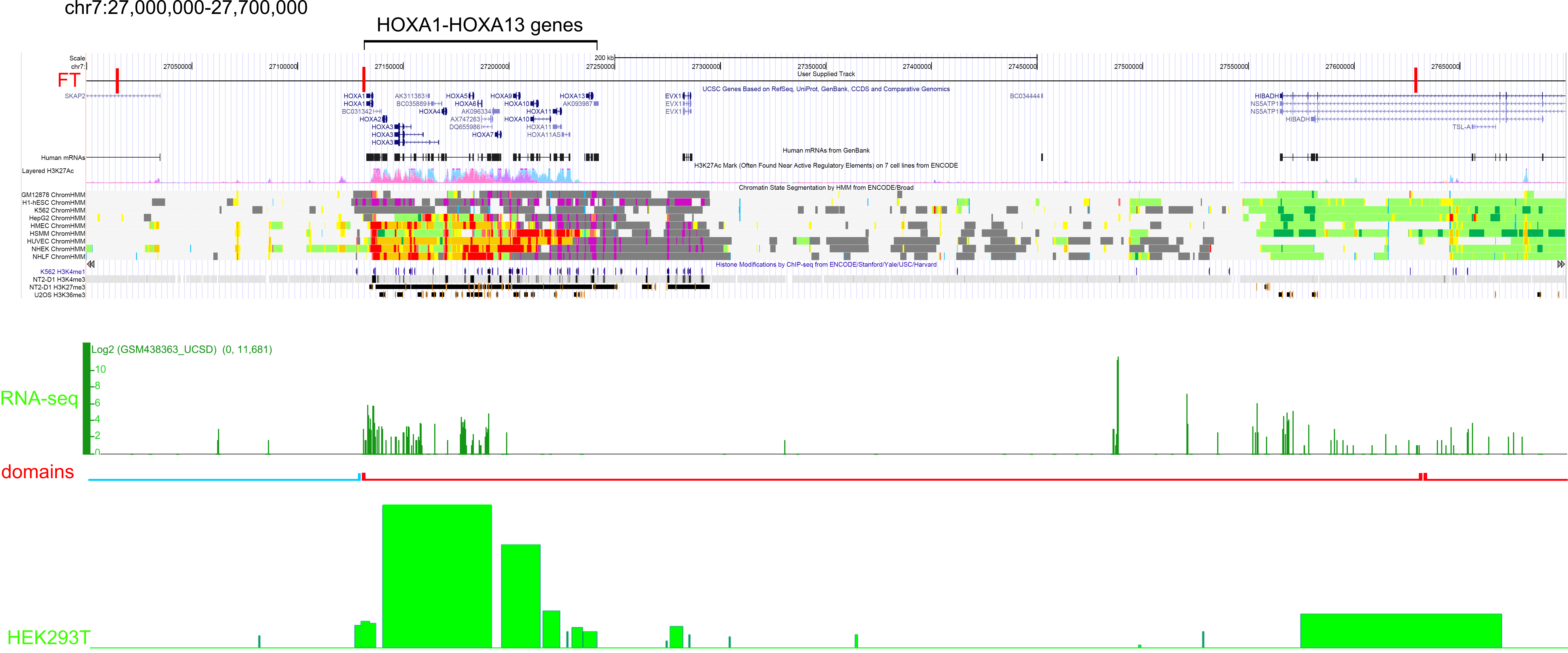

Supplement: Figure S13 — Coordinated expression inside the 498 kb forum domain that possesses the HOXA gene cluster. The UCSC Genome Browser on Human Feb. 2009 (GRCh37/hg19) Assembly was used. UCSC genes, Human mRNAs from GenBank, the H3K37Ac mark from Encode, chromatin state segmentation by HMM from Encode/Broad, and some histone modifications by ChIP-Seq from Encode are indicated. The “RNA-seq” lane corresponds to expression of mRNAs in IMR90 cells (GEO accession number GSM438363). Forum domain containing actively transcribed genes are indicated by the red bracket. “HEK293T” lanes correspond to the expression of mRNA in HEK293T cells (microarray data using Affymetrix Human Exon 1.0 ST expression arrays, wgEncodeEH002692_2). (TIF) [file pgen.1003429.s013.tif]

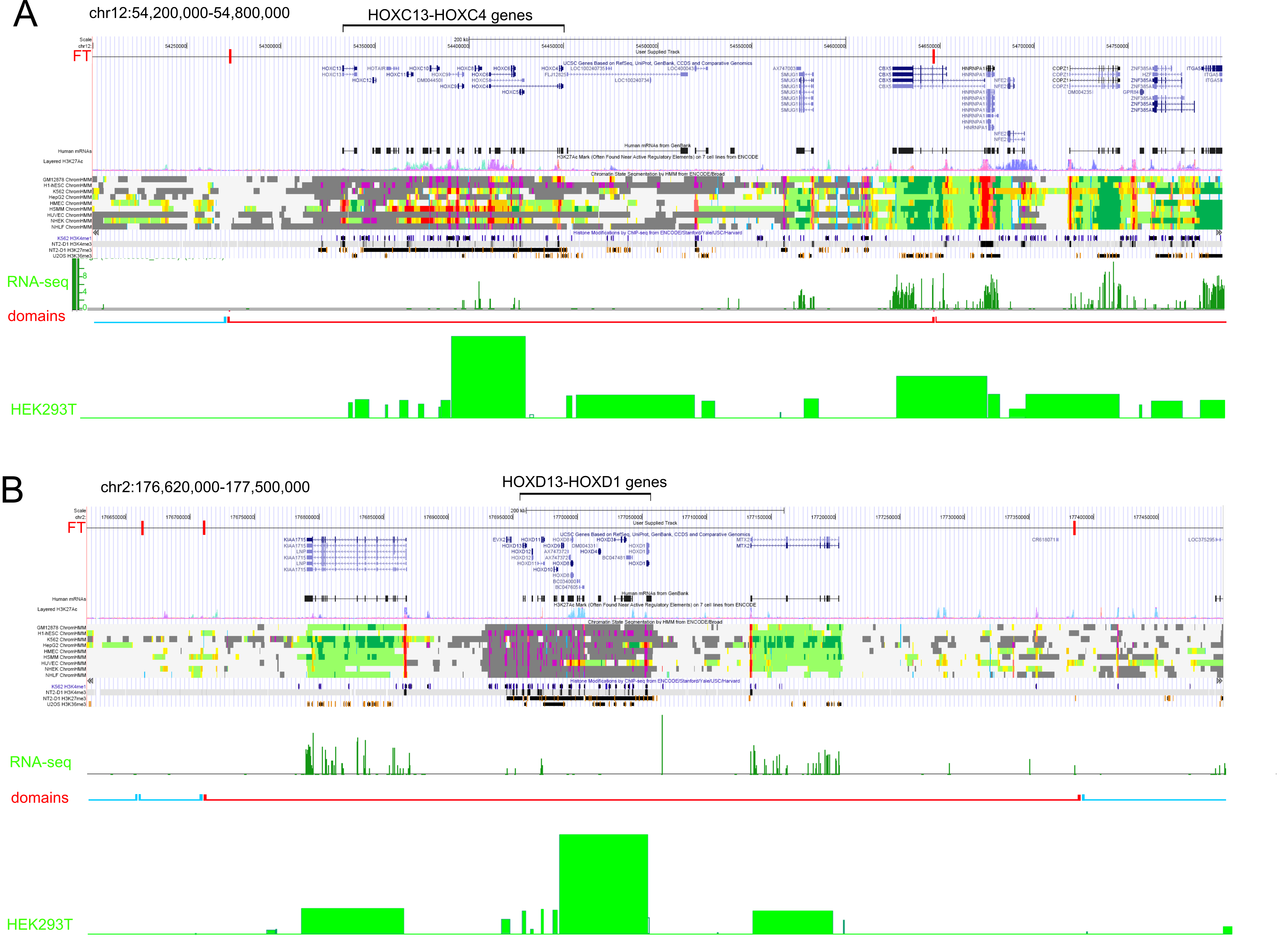

Supplement: Figure S14 — Coordinated expression inside the 374 kb forum domain that possesses the HOXC gene cluster (A) and inside the 673 kb forum domain that possesses the HOXD gene cluster (B). The UCSC Genome Browser on Human Feb. 2009 (GRCh37/hg19) Assembly was used. UCSC genes, Human mRNAs from GenBank, the H3K37Ac mark from Encode, chromatin state segmentation by HMM from Encode/Broad, and some histone modifications by ChIP-Seq from Encode are indicated. The “RNA-seq” lane corresponds to expression of mRNAs in IMR90 cells (GEO accession number GSM438363). Forum domains containing actively transcribed genes are indicated by the red bracket. “Domains” lanes indicate the forum domains containing the silent or weakly expressed genes (blue brackets). “HEK293T” lanes correspond to the expression of mRNA in HEK293T cells (microarray data using Affymetrix Human Exon 1.0 ST expression arrays, wgEncodeEH002692_2). (TIF) [file pgen.1003429.s014.tif]

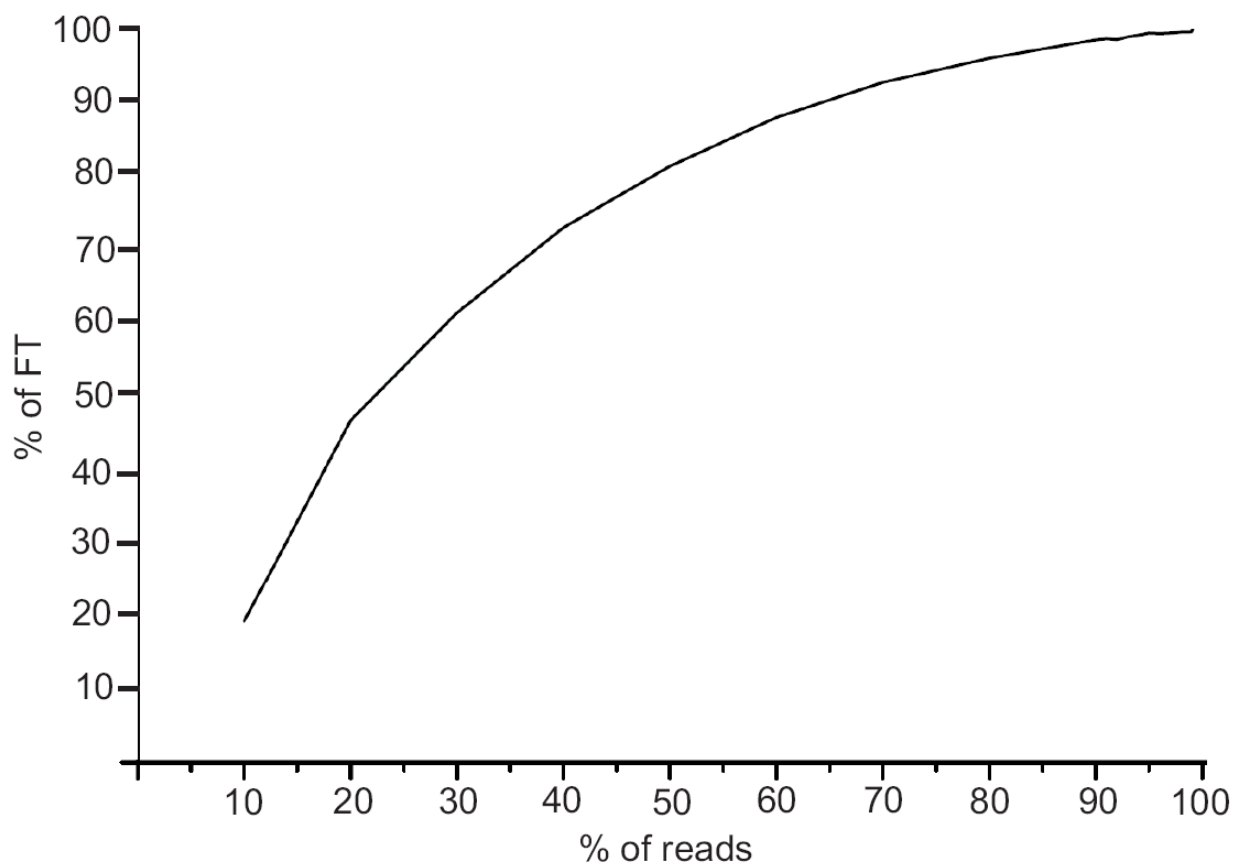

Supplement: Figure S15 — Saturation curve. % of FT plotted against decreasing % of reads reveals a plateau in the range between 90 and 100% of reads. The step was equal to 1% of reads in the range from 100% towards 70% and 5% down to 10% of reads. The curve indicates that practically all FT, corresponding to hot spots of DSBs, were defined. (PDF) [file pgen.1003429.s015.pdf]
