# Supplementary material for: DNA Double-Strand Breaks Coupled with PARP1 and HNRNPA2B1 Binding Sites Flank Coordinately Expressed Domains in Human Chromosomes
Source: PLoS Genet. 2013 Apr 4;9(4):e1003429. doi: 10.1371/journal.pgen.1003429 (PMC3616924; doi:10.1371/journal.pgen.1003429)

1090 bp: >hg19_dna range=chr16:79245075-79246164

AGTGGTTGGTTTGTCAGACAGAAGGTTCTACAAACGATTTGGTATCGAAT
TACATTATACATGGAGAAGTTGAGGGGTTGGATGAATTTGTTCTTGCACG
TTCAGAAGGATACCATCTTTTTCTCTGTGTGGAAGAGGCGCCTGCCACGA
CGTATTGATATGTGTATTTATTTTCCAAGCCTGTCCCTGTATGAGGTGTC Intron
AAAAGTTACACCAGCTTTACAAGCGGAGTTTATGAACTCGTTTTTCCAGG
ATAGTCACATTATACTTTTTACAGTCATGTGCTTTCAGCCCAGTACCCTT
TGCTATGCCAAGATCCAGCTGAAACTGAACCAGGTGGGGGAGGCCTGCTA
ATGCCCAGGCAGTCGAAATGACGCCATCTCATCACTCCTTTTCTTAAAAT
TTTTTTTTGTCTTTCTTCTTGGATTTCCAGCA

ACAGGGAGCTGCCACCAC
CGTGTACTGTGCTGCTGTCCCAGAACTGGAGGGTCTGGGAGGGATGTACT
TCAACAACTGCTGCCGCTGCATGCCCTCACCAGAAGCTCAGAGCGAAGAG
ACGGCCCGGACCCTGTGGGCGCTCAGCGAGAGGCTGATCCAAGAACGGCT
TGGCAGCCAGTCCGGCTAAGTGGAGCTCAGAGCGGATGGGCACACACACC
CGCCCTGTGTGTGTCCCCTCACGCAAGTGCCAGGGCTGGGCCCCTTCCAA 3’ Exon - coding sequence
ATGTCCCTCCAACACAGATCCGCAAGAGTAAAGGAAATAAGAGCAGTCAC
AACAGAGTGAAAAATCTTAAGTACCAATGGGAAGCAGGGAATTCCTGGGG
TAAAGTATCACTTTTCTGGGGCTGGGCTAGGCATAGGTCTCTTTGCTTTC
TGGTGGTGGCCTGTTTGAAAGTAAAAACCTGCTTGGTGTGTAGGTTCCGT
ATCTCCCTGGAGAAGCACCAGCAATTCTCTTTCTTTTACTGTTATAGAAT
AGCC**TGA**

GGTCCCCTCGTCCCATCCAGCTACCACCACGGCCACCACTGCA
GCCGGGGGCTGGCCTTCTCCTACTTAGGGAAGAAAAAGCAAGTGTTCACT 3’ Exon - UTS
GCTCCTTGCTGCATTGATCCAGGAGATAATTGTTTCATTC

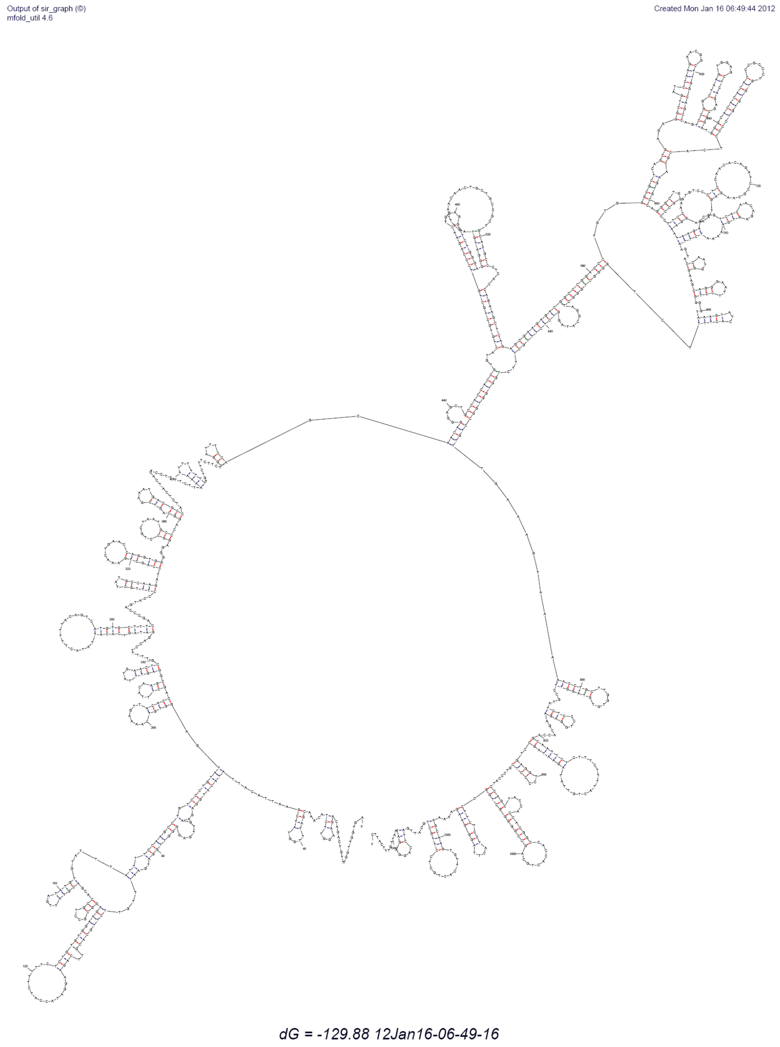

Supplement: Figure S4 — Z-DNA region and palindromes inside the 1090 bp sequence that possesses the mapped FT in the 3′ exon of the WWOX gene. This sequence escapes cloning in E. coli cells. The Z-DNA region (highlighted in yellow) was detected using ZHunt Online software (http://gac-web.cgrb.oregonstate.edu/zDNA/index). The region corresponding to FT reads is shown in blue (the Sau site is indicated in red). The underlined portion is a shorter amplified DNA fragment that also escapes cloning. Folding of the 1090 bp fragment was performed using the UNAFold Web Server http://mfold.rna.albany.edu/?q=mfold/DNA-Folding-Form). (PDF) [file pgen.1003429.s004.pdf]
