# Supplementary material for: DNA Double-Strand Breaks Coupled with PARP1 and HNRNPA2B1 Binding Sites Flank Coordinately Expressed Domains in Human Chromosomes
Source: PLoS Genet. 2013 Apr 4;9(4):e1003429. doi: 10.1371/journal.pgen.1003429 (PMC3616924; doi:10.1371/journal.pgen.1003429)
Supplement: Table S1 — Primers used in quantitative PCR experiments. (DOC) [file pgen.1003429.s016.doc]

Supporting Table S1. Primers used in quantitative PCR experiments.

| Region’s  Name | Primers | Sequences, 5’-3’ | FT reads coordinates,  hg19 | Reads#/  Length, bp |
| --- | --- | --- | --- | --- |
| FT-WWOX | pr(+) | AGCTGCCACCACCGTGTACTGT | chr16, 79,245,575.. 79,245,663 | 33/90 |
|  | pr(-) | ATCCGCTCTGAGCTCCACTTAG |  |  |
| FT-2 | FT-2 (+) | ATGTGGCTGTCTTGGTTTGAGTTT | chr1, 25,887,369.. 25,887,410 | 42/121 |
|  | FT-2 (-) | AGGGATATGAAAACGCCATTATTTAA |  |  |
| FT-4 | FT-4 (+) | CATAGGCTTATTTCAATATTTTAAAATAT | chr1, 109,305,338.. 109,305,373 | 36/16 |
|  | FT-4 (-) | AGGTACAGTACCCATATGGCCTT |  |  |
| FT-7 | FT-7 (+) | TATGTTTTGTGTCAAAGACTCACCT | chr1, 120,282,835.. 120,282,948 | 114/15 |
|  | FT-7 (-) | CACTGCCTCTCACTAACCCTGT |  |  |
| 5.8S rDNA-  non FT | pr(+) | CGGTGGATCACTCGGCTCGT | Non FT  chr16, 79,225,576- 79,225,859 | - |
|  | pr(-) | GCCGCAAGTGCGTTCGAAGTG |  |  |
| WWOX-non FT | pr(+) | TAGGCCATGATAAAGAATAAATACC | Non FT | - |
|  | pr(-) | ATCACCTGATGTGTGGTGAGCC |  |  |
