# Supplementary material for: DNA Double-Strand Breaks Coupled with PARP1 and HNRNPA2B1 Binding Sites Flank Coordinately Expressed Domains in Human Chromosomes
Source: PLoS Genet. 2013 Apr 4;9(4):e1003429. doi: 10.1371/journal.pgen.1003429 (PMC3616924; doi:10.1371/journal.pgen.1003429)
Supplement: Table S2 — Primers used in the ChIP experiments shown in Figure 9C. (DOC) [file pgen.1003429.s017.doc]

Supporting Table S2. Primers used in the ChIP experiments shown in Figure 9C.

| Region’s  Name | Primers | Sequences, 5’-3’ |
| --- | --- | --- |
| FT-WWOX  upstream | pr(+) | AGCTGCCACCACCGTGTACTGT |
|  | pr(-) | CCCAGACCCTCCAGTTCTGGGA |
| FT-WWOX  downstream | pr(+) | GCTTGGCAGCCAGTCCGGCTAA |
|  | pr(-) | ATCCGCTCTGAGCTCCACTTAG |
| FT-4  upstream | pr(+) | CATAGGCTTATTTCAATATTTTAAAATAT |
|  | pr(-) | GGATATTAAGTGAGCAGTTCTTCAGC |
| FT-4  downstream | pr(+) | GTAGTATAATGAATGTGACACTGCTT |
|  | pr(-) | AGGTACAGTACCCATATGGCCTT |
