# Supplementary material for: DNA Double-Strand Breaks Coupled with PARP1 and HNRNPA2B1 Binding Sites Flank Coordinately Expressed Domains in Human Chromosomes
Source: PLoS Genet. 2013 Apr 4;9(4):e1003429. doi: 10.1371/journal.pgen.1003429 (PMC3616924; doi:10.1371/journal.pgen.1003429)
Supplement: Table S3 — Percent of low expressing or silent forum domains in four cell lines. Names of cell lines and their corresponding accession numbers are indicated. The conversion of data for the second and third columns from hg18 to hg19 coordinates was performed using the LiftOver program (http://hgdownload.cse.ucsc.edu/admin/exe). The median values of transcription levels in coding regions (representing exon array signals) within a particular forum domain were used. The average expression level of forum domains in a particular chromosome was determined as a sum of expression data per chromosome divided into domains numbers. The values in the last four columns correspond to % of low expressing (below the average expression level in a particular chromosome) or silenced forum domains in a corresponding chromosome. Most domains in different cell lines are silent or expressed at very low levels. (DOC) [file pgen.1003429.s018.doc]

Supporting Table S3. Percent of silent forum domains in different cell types.

| **Chromosome** | **IMR90 cell line, fetal lung fibroblasts,**  **GSM438363** | **K-562 cell line, pleural cells,**  **GSM922955** | **embryonic stem cells,**  **GSM572173** | **HEK293T cells,**  **wgEncodeEH002692_2** |
| --- | --- | --- | --- | --- |
| 1 | 87 | 86 | 80 | 72 |
| 2 | 89 | 86 | 87 | 71 |
| 3 | 81 | 90 | 79 | 72 |
| 4 | 81 | 84 | 94 | 72 |
| 5 | 87 | 82 | 89 | 74 |
| 6 | 80 | 82 | 76 | 73 |
| 7 | 86 | 87 | 79 | 73 |
| 8 | 83 | 91 | 83 | 72 |
| 9 | 78 | 86 | 77 | 75 |
| 10 | 82 | 77 | 79 | 72 |
| 11 | 84 | 82 | 77 | 74 |
| 12 | 81 | 87 | 75 | 72 |
| 13 | 89 | 89 | 80 | 77 |
| 14 | 82 | 89 | 77 | 74 |
| 15 | 82 | 82 | 75 | 70 |
| 16 | 76 | 82 | 76 | 72 |
| 17 | 84 | 86 | 76 | 75 |
| 18 | 76 | 87 | 75 | 70 |
| 19 | 78 | 78 | 74 | 68 |
| 20 | 79 | 84 | 79 | 71 |
| 21 | 92 | 88 | 77 | 73 |
| 22 | 75 | 83 | 77 | 70 |
| X | 86 | 95 | 91 | 78 |
| Y | 90 | N/A | 90 | 87 |
