# Supplementary material for: DNA Double-Strand Breaks Coupled with PARP1 and HNRNPA2B1 Binding Sites Flank Coordinately Expressed Domains in Human Chromosomes
Source: PLoS Genet. 2013 Apr 4;9(4):e1003429. doi: 10.1371/journal.pgen.1003429 (PMC3616924; doi:10.1371/journal.pgen.1003429)
Supplement: Text S1 — Extended Materials and Methods. (DOC) [file pgen.1003429.s019.doc]

**Text S1**

**This file includes**

**Extended Materials and Methods**

**Supporting References**

**Extended Materials and Methods**

**Preparation of DNA-agarose plugs**

DNA-agarose plugs were prepared as described by Tchurikov and Ponomarenko, 1992. About 6 million HEK293T cells in 2 mL of culture medium were pelleted by centrifugation at 2000 rpm, resuspended in 0.3 mL of the same medium, gently mixed at 42 °C with an equal volume of a 1% agarose L (LKB) in DMEM, and distributed in a mold containing 100-L wells. The mold was placed on ice for 2–5 min and covered with Parafilm. The agarose plugs were then placed in Petri dishes with 5 mL of solution containing 0.5 M EDTA (pH 9.5), 1% sodium laurylsarcosine, and 1–2 mg of proteinase K solution per mL for 40–48 h at 50 °C. They were stored at 4 °C in the same solution. Each DNA-agarose plus usually contained about 15 μg of DNA corresponding to about 1 million cells.

To test the quality of isolated DNA, fractionation in PFGs was performed as described previously (Tchurikov and Ponomarenko, 1992). Portions of the original agarose-DNA plugs (5–50 μL) containing 1–10 μg of DNA were used for electrophoresis without any restriction enzyme digestion. The DNA samples were run in 0.8% agarose gels on an LKB Pulsaphor system using a hexagonal electrode and switching times of 25, 100, or 450 sec.

For elution of DNA preparations, fractionation in a 1% agarose conventional mini-gel was performed. One-half of a DNA-agarose plug was washed in 1 x TE three times (for 15 min each) followed by washing three times in the same solution containing 17.4 μg/mL PMSF in ethanol. After fractionation in the mini-gel, the ethidium-bromide stained DNA band was excised and electroeluted inside a cellulose membrane dialysis bag. After overnight dialysis without stirring against 1 l of 0.01 x TE at 4 °C, the DNA was concentrated with PEG (4 °C) and redialyzed.

**RAFT procedure**

Rapid Amplification of Forum domains Termini (RAFT) was performed as described by Tchurikov et al., 2011. About 1.5 μg of isolated DNA (see above) were treated by Klenow fragment of *E.coli* DNA polymerase I and then ligated with 70 ng of double-stranded oligonucleotide (25-bp-long 5’-phosphorylated 5’ pCCCCTGCAGTATAAGGAGAATTCGGG 3’ oligonucleotide annealed with 26-bp-long 5’ biotinylated 5’ bio-CCGAATTCTCCTTATACTGCAGGGG 3’ oligonucleotide) in 150 μL of solution containing 0.1 M NaCl, 50 mM Tris HCl (pH 7.4), 8 mM MgCl_2_, 9 mM 2-mercaptoethanol, 7 μM ATP, 7.5% PEG, and 40 units of T4 DNA ligase at 20 °C for 16 h. After heating at 65 °C for 10 min, the DNA preparation was digested with Sau3A enzyme to shorten the forum domain to the termini attached to the ligated oligonucleotide. The selection of such termini was performed in 0.5 mL Eppendorf tubes using 300 μL of suspension containing Streptavidin MagneSphere Paramagnetic Particles (SA-PMP, Promega, Madison, WI, USA) according to the manufacturer’s recommendations. After extensive washing with 0.5xSSC to remove DNA fragments corresponding to internal parts of forum domains, the forum termini (FT) DNA preparation was eluted from the SA-PMP using digestion with *Eco*RI enzyme in a final volume of 50 μL (double-stranded FT). The FT were then ligated with 100x molar excess of double-stranded Sau3A adaptor (5’-phosphorylated 5’ pGATCGTTTGCGGCCGCTTAAGCTTGGG 3’ oligonucleotide annealed with 5’ CCCAAGCTTAAGCGGCCGCAAAC 3’ oligonucleotide). In some experiments the FT DNA preparation was eluted from the SA-PMP using heating via incubation at 100 °C for 3 min in 50 μL of 0.01xTE (single-stranded FT). Before heating, the FT preparation was ligated with 100x molar excess of double-stranded Sau3A adaptor in suspension with SA-PMP (see above). Both final DNA samples (double-stranded FT or single-stranded FT) were used for PCR amplifications. Forty cycles of PCR amplification in 30 μL of a solution containing 67 mM Tris HCl (pH 8.4); 6 mM MgCl2; 10 mM 2-mercaptoethanol; 16.6 mM ammonium sulphate; 6.7 μM EDTA; 5 μL 5 mg/mL BSA; 1 mM dNTPs; 1 μg of primer corresponding to the Sau3A adaptor (5’ CCCAAGCTTAAGCGGCCGCAAAC 3’); 1 μg of primer corresponding to the biotinylated oligonucleotide (5’ CCGAATTCTCCTTATACTGCAGGGG 3’), and 1 u of Taq (or Pfu) polymerase were performed using Eppendorf Mastercycler Personal. Amplification conditions were 90 °C for melting, 65 °C for annealing, and 72 °C for extension, for 1 min each.

**FISH**

Total DNA isolated from HEK 293T cells and RAFT preparations (4 μg each) were labeled with Alexa Fluor 5 and Alexa Fluor 3, respectively, using a BioPrime total genomic labeling system (Invitrogen) according to the manufacturer’s recommendations. The specific activities measured using a [NanoDrop 2000 Spectrophotometer](http://www.nanodrop.com/Productnd2000overview.aspx) were equal to 1.03 and 0.89 pmol/ng for the total DNA and RAFT probes, respectively.

G-banding was performed prior to FISH using a standard procedure (Seabright, 1971). Metaphases were photographed, and slides were de-stained in methanol and fixed with 0.5% formaldehyde. FISH was performed using a standard protocol (Yang et al., 1999; Graphodatsky et al., 2000). For each hybridization experiment we used 1 µg each of Alexa3-RAFT and Alexa5-total DNA probes in 20 µL of hybridization buffer (10% dextran sulphate, 50% formamide, 2xSSC). Paints were denatured at 96 ˚C for 3 min and re-annealed for 30 min at 42 ˚C. Probes were hybridized overnight at 42 ˚C. Post-hybridization washes included 50% formamide, 2xSSC (three times), 2xSSC (three times, 5 min each). Chromosomes were counterstained with DAPI. Images were captured using In Situ Imaging System (ISIS) software (Metasystems) by a Metasystems CCD camera mounted on an Axioplan 2 imaging (Zeiss) microscope. The microscope was equipped with filter sets for DAPI, Cy3, and Cy5. All image processing was performed using PaintShop Photo Pro X2 (Corel).

**Nuclear protein extracts**

Nuclear proteins from HEK293T cells were isolated as described by Andrews and Faller, 1991. The final protein concentration measured using a [NanoDrop 2000 Spectrophotometer](http://www.nanodrop.com/Productnd2000overview.aspx) was about 1–3 μg/μL.

**Isolation of proteins binding with RAFT preparations**

The RAFT preparation was amplified as described above using biotinylated 5’ CCGAATTCTCCTTATACTGCAGGGG 3’ primer and Taq polymerase. Initially, 150 μL of nuclear protein extract (150–300 μg of proteins) were pre-exhausted for 10 min at 12 ºC in 400 μL of solution containing 20 mM HEPES, pH 7.6, 4% Ficoll, 5 mM MgCl_2_, 0.2 mM EDTA, 1 mM DTT, 30 μg of poly(I/C), and 30 μg of poly(dI/dC). In some experiments, up to 25 μg of non-biotinylated RAFT preparation (specific competitor) or up to 30 μg of 300 bp PCR amplified DNA fragment corresponding to the *Col*E1-derived replication origin sequence (2251 to 2550 bp region in pGL3-Enhancer vector, Promega) (non-specific competitor) were added. After pre-incubation, the mixture containing the pre-exhausted nuclear protein extract was added to 0.4 μg of biotinylated RAFT preparation bound with 200 μg of SA-PMP (Promega), and incubation at 12 ºC was carried out for 1 h with gentle mixing every 10 min. The binding proteins were selected on SA-PMP (Promega) according to the manufacturer’s recommendations, but we performed five washings with 100 μL of the buffer containing 20 mM HEPES, pH 7.6, 4% Ficoll, 5 mM MgCl_2_, 0.2 mM ETDA, and 1 mM DTT. Finally, the binding proteins were eluted from SA-PMP in 50 μL of 0.5 M NaCl. The selected proteins were fractionated in 5–18% SDS-PAGE using Broad Range Protein Molecular Weight Markers (Promega). Silver-staining was performed using a ProteoSilver Plus Kit (Sigma). The bands of interest were excised and used for a mass spectrometry.

**Trypsin digestion and mass spectrometry**

The protein spots were excised from the PAAG and digested with trypsin. The silver-stained protein spots (1–2 mm^3^) were de-stained with 20 μL of solution containing 15 mM potassium ferricyanide and 50 mM sodium thiosulfate and were washed twice with 100 μL of Millipore-Q water. The gel pieces were dehydrated with 40 μL acetonitrile (ACN) and rehydrated with 2 μL of a digestion solution containing 20 mM ammonium bicarbonate and 10 ng/μL sequencing grade trypsin (Promega). Digestion was carried out overnight at 37 °C. Peptides were extracted with 4 μL of 0.5% trifluoroacetic (TFA) acid solution. To obtain the peptide mass fingerprint, 2 μL of extract were mixed with 0.5 μL of 2,5-dihydroxybenzoic acid saturated solution in 20% ACN and 0.5% TFA on the stainless steel MALDI sample target plate. Mass spectra were recorded using an Ultraflex II MALDI-TOF/TOF mass spectrometer (Bruker Daltonics, Billerica, MA, USA) equipped with a Nd laser (354 nm). The MH+ molecular ions were detected in reflector mode in the mass range 700–4000 m/z. The accuracy of the mass peak measurement after internal calibration with the peaks of trypsin autolysis was 50 ppm. Proteins were identified from peptide fingerprints; the search was conducted using Mascot software (Matrix Science). The US National Center for Biotechnological Information ([http://www.ncbi.nlm.nih.gov](http://www.ncbi.nlm.nih.gov/)) database was used for the search; possible modifications of cysteine residues by acrylamide and methionine oxidation were taken into consideration. The cut off was 84 (p < 0.05).

**Computer treatments**

Raw data in SFF format were obtained using a 454 Roche GS FLX Life Sciences pyrosequencing machine. Data then were decoded to FASTQ/FASTA format using PyroBayes (<http://bioinformatics.bc.edu/marthlab/PyroBayes>). PyroBayes software was designed to assign more accurate base quality estimates of the 454 pyrosequences compared with the proprietary Roche software (Quinlan et al., 2008). The next step was to cut off two primers: primer_HindIII_NotI – CCCAAGCTTAAGCGGCCGCAAAC and primer_EcoRI_PstI – CCGAATTCTCCTTATACTGCAGGGG. This procedure requires as accurate as possible pair alignment because of the significant number of errors in the data produced by the pyrosequencing machine. FASTA version 34.26.5 (<http://faculty.virginia.edu/wrpearson/fasta>) was used for this task with the parameters "-z 11,12,14,15 -A -E 0.01 -n -m 9 -Q" to turn off as much of FASTA’s own logic as possible because it produces unstable and even quite different results between different runs of the program with the same data. As a result, primer’s cut off was performed by Perl script using BioPerl as interface to FASTA, with the assumption that a primer should be at the any end of a read, but not further than 5 bp from it.

All sequences shorter than 18 bp were removed from the dataset. The final mapping was performed using BWA ([http://bio-bwa.sourceforge.net](http://bio-bwa.sourceforge.net/)) and samtools ([http://samtools.sourceforge.net](http://samtools.sourceforge.net/)) with the *Homo sapiens* masked genome (assembly GRCh37p5/hg19) as the database (taken in the form of MFA files from <ftp://ftp.ncbi.nih.gov/genomes/H_sapiens/Assembled_chromosomes/seq>). The mapping result was deposited into the GEO database with the accession ID GSE35065 (<http://www.ncbi.nlm.nih.gov/geo/query/acc.cgi?acc=GSE35065>). The original 357,893 reads were mapped into 75,794 FASTA unique genomic regions each containing from one to 456 aligned reads corresponding to a region. Then we selected 17,307 “provisional FT”, representing the regions to which at least two overlapping reads corresponded. But the computed DNA fragments that could be produced by these 17,307 provisional FT revealed the median of DNA profile that was smaller than was really observed in the pulsed-field gels.

We used 454 sequencing producing rather long reads. That is why we were able to observe that the most part of these “provisional FT” has the focused DSBs, i.e. individual DSBs were found to be located closely to each other. As far as the 5’ biotinylated oligos were ligated to the sites of DSBs, the fact strongly suggests that the selected reads correspond to independent breaks occurring closely to each other in the same genomic region and also indicates the non-randomness of breaks. That is why we decided to use a cut-off based on the overlapping value to select more focused DSBs. We used the 1.8 cut-off value and selected regions to which the reads overlapping at least 80% of their length (1+0.8 = 1.8 threshold) corresponded. Using this cut-off we slightly reduced the amount of FT - from 17,307 to 16,535. Only after this cut-off we observed the perfect correspondence of computed and real DNA profiles (Figure 1A, C). It follows that to each of 16,535 genomic regions, representing the finally selected FT, at least two 80% overlapping reads with close to each other located DSBs correspond. These reads are presented in .gff and .wig files, which are divided by chromosomes for convenience. The data in the .gff and .wig files are the same; only the format differs. The saturation curve shown in Figure S15 indicates that practically all FT were defined.

All domain-gene comparisons and statistical evaluations were performed with the gene’s database from the same GRCh37p5 genome build (taken in the form of GBS files from <ftp://ftp.ncbi.nih.gov/genomes/H_sapiens/Assembled_chromosomes/gbs>) by the Perl script with BPLITE module as interface for GBK/GBS files.

We used Monte-Carlo simulations to evaluate the probability of the random occurrence of the same amount of FT (consisting of overlapping reads) in the *FHIT* gene. This procedure was performed as follows. *FHIT* is located on chromosome 3. Chromosome 3 contains 4628 clusters of reads corresponding to 1005 FT (each overlapping at least by 0.8 of their length - 1.8 reads value). FHIT itself contains 7 FT (33 reads), and adjacent regions (+/- 500kb) contain 11 FT (57 reads). FT size (in bp) for chromosome 3 has the following statistics: mean value = 61, median = 50, and [5%, 95%] percentile = [32, 129]. We accepted the upper 95% percentile FT size as being equal to 129 (which is the worst case in 95% of cases) for our studies. Random breaks were generated in the same quantities and within the same limits as those actually found in chromosome 3 using the Mersenne Twister pseudorandom values generator. We then analyzed the pattern of FT in the area of interest and considered only the randomly created “FT” that overlapped by 0.8 by their length or more (equivalent to 1.8 reads value in the summary table). These simulations were run up to 10,000,000 times and then stopped. After ten runs of the simulation (totalling 100,000,000 runs), we never encountered more than five simultaneously created random overlapping breaks in the region. Therefore, we defined the upper probability of seven overlapping breaks randomly emerging simultaneously to be lower than 10^-7^, or 0.00001%. Similarly, simulations were performed for the *WWOX* gene.

The circular permutation approach (Cabrera et al., 2012) was used to test if genes in the same forum domain are more similarly expressed than genes in different domains. We determined the median values of gene expression levels for each gene using the HEK293T expression data (wgEncodeEH002692_2) and the human genes database from GBS files hs_ref from NIH (<ftp://ftp.ncbi.nlm.nih.gov/genomes/H_sapiens/Assembled_chromosomes/gbs>). The huge volume of the data prompted us to employ the program using C language which allowed the acceptable speed of calculations and the volumes of memory.

For the study 5,732 forum domains (from 16,535 total domains number) possessing at least two genes (or one gene and more than a half of another gene) were selected. For each domain initially we calculated the mean values of all possible pair-wise expressions |E_a_-E_b_|, i.e. Δ*_D_*=<|E_a_-E_b_|>, then obtained the average data per domain in the whole genome, calculating the mean value of Δ*_D_* genome-wide, *δ_D_*= <<|E_a_-E_b_|>>, where E_a_ and E_b_ are the median values of expression levels of genes located inside a forum domain.

We used the circularly shifting by random value of gene median expression values between genes. We consider the genome to be "circular" and ordered in chromosomes from chromosome 1 to chromosome Y and restarting from chromosome 1 again (Cabrera et al. 2012) to save the pattern of genes and domains. The random shift in the median values of expression level for each gene to the random *n* gene number of genes to the left or to the right (in clockwise or counter clockwise direction) on the reference genome were performed by rotation with respect to their genome location, where *n* was less or equal to the total genes numbers in the genome (from 1 up to 28563). Thus, the genes located inside a forum domain retained the same position with respect to each other but, at each shift, gained new random median values of expression levels for each gene.

Then we performed the same calculations for permutated (by circular shifting) expression data - *δ_shift_*. To test the difference in mean values of gene expression levels in each forum domain upon permutations we used a standard normal deviate, z [Gauss criterion](http://www.encyclopediaofmath.org/index.php/Gauss_criterion), which we calculated using the variance values of Δ*_D_* and Δ*_shift_*. The z [Gauss criterion](http://www.encyclopediaofmath.org/index.php/Gauss_criterion) for D number of forum domains thus will be:

$$z=\frac{\delta_{D}-\delta_{shift}}{\sqrt{\frac{\sigma_{D}^{2}}{D}+\frac{\sigma_{shift}^{2}}{D}}}$$

We performed 10,000 permutation experiments calculating Z values for each experiment. To test the acceptability of the Z test we used the Anderson-Darling test for normality. It was described that Shapiro-Wilk test has the best power for a given significance, followed closely by Anderson-Darling when comparing the Shapiro-Wilk, Kolmogorov-Smirnov, Lilliefors, and Anderson-Darling tests (Razali and Wah, 2011). Keeping in mind that Shapiro-Wilk test has a limit up to 5,000 values in the vector, while we have 5,732 domains, we selected the Anderson-Darling test and obtained the test for normality for all cases with an error p<2.2E-16, indicating that Δ*_shift_* is distributed normally indeed so we can use z criterion.

Simulations were performed by *ad hoc* program using Perl with interface to R language with library nortest, from which the we used ad.test() for the test for normality. The results were as follows: z mean=-6.02343, z median=-6.0169, z max=-4.15898, indicating that at least |z|>4, and p-value<0.0001. The data strongly suggest that expression levels were much more similar between genes within the forum domains than between a random stretches of the same number of adjacent genes in the genome.

**Quantitative real-time PCR analysis of DSBs inside the 3’ exon of the *WWOX* gene, FT-2, FT-4, and FT-7**

To estimate the amount of damaged DNA molecules that possesses the mapped FT inside the 3’ exon of the *WWOX* gene (Figure 3E, F), we performed real-time PCR across the FT using Applied Biosystems’ 7500 Real-Time PCR System. DNA preparations from HEK 293T cells were isolated in solution by a procedure that simulates the isolation of DNA in DNA-agarose plugs. About 1.5 x 10^6^ cells in 500 μL of DMEM containing 10% FBS were precipitated in 1.5 mL Eppendorf tubes in a minispin centrifuge (1500 rpm for 2 min) at room temperature. They then were suspended in 50 μL of the same medium at 42 ºC. For isolation of control DNA (“intact”), the suspension of cells was immediately mixed with 100 μL of solution containing 0.5 M EDTA (pH 9.5), 1% sodium laurylsarcosine, and 2 mg of proteinase K solution per mL. After incubation of the mixture for 48 h at 50 °C, the control DNA was isolated by phenol-chloroform extractions followed by isopropanol precipitation. To isolate forum domains, the suspension of cells in 50 μL of DMEM containing 10% FBS after 2–3 min incubation at 42 ºC was additionally incubated for 5 min at 0 ºC, and then DNA was isolated as described above for the control DNA. The 4.8S ribosomal gene was used as a reference sequence for quantitative PCR. The following primers were used in real-time PCR: 5’ cgtgtactgtgctgctgtcccA 3’ and 5’ TcTGAGCTCCACTTAGCCGGAC 3’ (for the *WWOX* gene); and 5’ CGGTGGATCACTCGGCTCGT 3’ and 5’ GCCGCAAGTGCGTTCGAAGTG 3’ (for the 5.8S ribosomal gene). The amplified *wwox* and 5.8S DNA fragments were 182 bp and 125 bp in length, respectively. To build a standard curve, the amplified 1090 bp *WWOX* fragment (Figure S4) was used. Figure S5 shows that about 22% of molecules in the forum DNA preparation were damaged. The same results were obtained in four independent experiments with four different DNA preparations. Similarly, we performed quantitative real-time PCR experiments across three other FT: FT-2, FT-4, and FT-7. The corresponding primers and coordinates of the FT are indicated in Table S3. In the experiments, Hydroxyurea (HU) HEK 293T cells were incubated with 2 mM HU for 18 h, as described by Gagou et al. (2010), and then used for DNA preparations (“intact” DNA and forum domains). Heat shock treatment of HEK 293T cells was performed by incubation at 43 °C for 20 min followed by incubation at 37 °C for 2.5 h, as described by Sonna et al. (2002).

**Supporting References**

Andrews, N.C., Faller, D.V. (1991). A rapid micropreparation technique for extraction of DNA-binding proteins from limiting numbers of mammalian cells. Nucl. Acids Res. *19*, 2499.

Gagou, M.E., Zuazua-Villar. P,, Meuth, M. (2010). [Enhanced H2AX phosphorylation, DNA replication fork arrest, and cell death in the absence of Chk1.](http://www.ncbi.nlm.nih.gov/pubmed/20053681) Mol. Biol. Cell. *21*,739-752.

[Graphodatsky, A.S](http://www.ncbi.nlm.nih.gov/pubmed?term=Graphodatsky AS%5BAuthor%5D&cauthor=true&cauthor_uid=10828614)., [Sablinam O,V](http://www.ncbi.nlm.nih.gov/pubmed?term=Sablina OV%5BAuthor%5D&cauthor=true&cauthor_uid=10828614),, [Meyer, M.N](http://www.ncbi.nlm.nih.gov/pubmed?term=Meyer MN%5BAuthor%5D&cauthor=true&cauthor_uid=10828614)., [Malikov, V.G](http://www.ncbi.nlm.nih.gov/pubmed?term=Malikov VG%5BAuthor%5D&cauthor=true&cauthor_uid=10828614)., [Isakova, E.A](http://www.ncbi.nlm.nih.gov/pubmed?term=Isakova EA%5BAuthor%5D&cauthor=true&cauthor_uid=10828614)., [Trifonov, V.A](http://www.ncbi.nlm.nih.gov/pubmed?term=Trifonov VA%5BAuthor%5D&cauthor=true&cauthor_uid=10828614)., [Polyakov, A.V](http://www.ncbi.nlm.nih.gov/pubmed?term=Polyakov AV%5BAuthor%5D&cauthor=true&cauthor_uid=10828614)., [Lushnikova, T.P](http://www.ncbi.nlm.nih.gov/pubmed?term=Lushnikova TP%5BAuthor%5D&cauthor=true&cauthor_uid=10828614)., [Vorobieva, N.V](http://www.ncbi.nlm.nih.gov/pubmed?term=Vorobieva NV%5BAuthor%5D&cauthor=true&cauthor_uid=10828614)., [Serdyukova, N.A](http://www.ncbi.nlm.nih.gov/pubmed?term=Serdyukova NA%5BAuthor%5D&cauthor=true&cauthor_uid=10828614)., *et al*. (2000). Comparative cytogenetics of hamsters of the genus *Calomyscus*. *Cytogenet.* Cell Genet. *88*, 296-304.

Matsumoto, M., Nishimura, T. (1998). Mersenne twister: a 623-dimensionally equidistributed uniform pseudo-random number generator. ACM Transactions on Modeling and Computer Simulation *8*, 3-30.

Seabright, M. (1971). A rapid banding technique for human chromosomes. Lancet *2*, 971-972.

Sonna L.A., Gaffin, S.L., Pratt, R.E., Cullivan, M.L., Angel, K.C., Lilly, C.M. (2002). [Effect of acute heat shock on gene expression by human peripheral blood mononuclear cells.](http://www.ncbi.nlm.nih.gov/pubmed/11960976) J. Appl. Physiol. *92*, 2208-2220.

Tchurikov, N.A., Ponomarenko, N.A. (1992). Detection of DNA domains in *Drosophila*, human and plant chromosomes possessing mainly 50- to 150-kilobase stretches of DNA. Proc. Natl. Acad. Sci. USA *89*, 6751-6755.

Tchurikov, N.A., Kretova, O.V., Sosin, D.V., Zykov, I.A., Zhimulev, I.F., Kravatsky Y.V. (2011). Genome-wide profiling of forum domains in Drosophila melanogaster. Nucl. Acids Res. 39, 3667-3685.

Quinlan, A.R., Stewart, D.A., Strömberg, M.P,   Marth, G.T. (2008). Pyrobayes: an improved base caller for SNP discovery in pyrosequences. Nat. Methods *5*, 179-181.

Yang, F., O`Brien, P.C., Milne, B.S. (1999). A complete comparative chromosome map for the dog, red fox, and human and its integration with canine genetic maps. Genomics *62*, 189-202.

Cabrera, C.P., Navarro, P., Huffman, J.E., Wright, A.F., Hayward, C., Campbell, H., Wilson, J.F., Rudan, I., Hastie, N.D., Vitart, V., Haley, C.S. (2012). [Uncovering networks from genome-wide association studies via circular genomic permutation.](http://www.ncbi.nlm.nih.gov/pubmed/22973544) G3 (Bethesda). *2*, 1067-1075. doi: 10.1534/g3.112.002618.

Razali, N., Wah, Y. (2011). Power comparisons of Shapiro-Wilk, Kolmogorov-Smirnov, Lilliefors and Anderson-Darling tests. J. Statistical Modeling and Analytics. *2*, 21–33.
